# Supplementary material for: Mebendazole for Differentiation Therapy of Acute Myeloid Leukemia Identified by a Lineage Maturation Index
Source: Sci Rep. 2019 Nov 14;9:16775. doi: 10.1038/s41598-019-53290-3 (PMC6856101; doi:10.1038/s41598-019-53290-3)
Supplement: Supplementary file 1 — Supplementary Figures and Tables [file 41598_2019_53290_MOESM1_ESM.pdf]

Supplementary Information:

Mebendazole for Differentiation Therapy of Acute Myeloid Leukemia Identified by a Lineage Maturation Index

Yulin Li[1, 2, 3] *, Daniel Thomas[4], Anja Deutzmann[3], Ravindra Majeti[4], Dean W. Felsher[3], David L. Dill[5]

[1]Center for Immunotherapy Research, Houston Methodist Research Institute, Houston, TX 77030; [2]Department of Medicine, Weill Cornell Medical College, New York, NY 10065; [3]Division of Oncology, Department of Medicine and Pathology, Stanford University School of Medicine; [4]Division of Hematology, Department of Medicine, and Institute for Stem Cell Biology and Regenerative Medicine, Stanford University School of Medicine; [5]Department of Computer Science, Stanford University, Stanford, CA 94305, USA.

*** Corresponding author**: Yulin Li, Houston Methodist Research Institute. 6670 Bertner Avenue. Houston, TX 77030. Phone: 713-441-7350; Fax: 713-441-7438; Email: yli@houstonmethodist.org

The supplemental information includes:
Supplementary Figure S1-S4.
Supplementary Table S1-S2.

Supplementary Fig S1

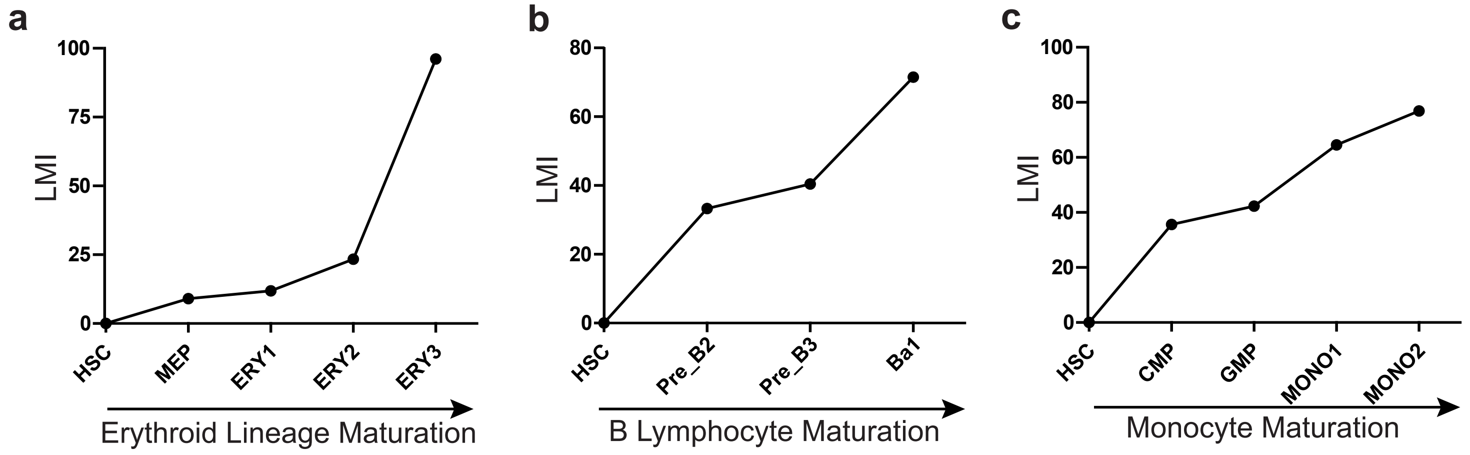

LMI can differentiate among various intermediate populations within normal human hematopoietic system. (a) human erythroid lineage. (b) human B lymphocyte lineage. (c) human monocytic lineage.

Supplementary Fig S2

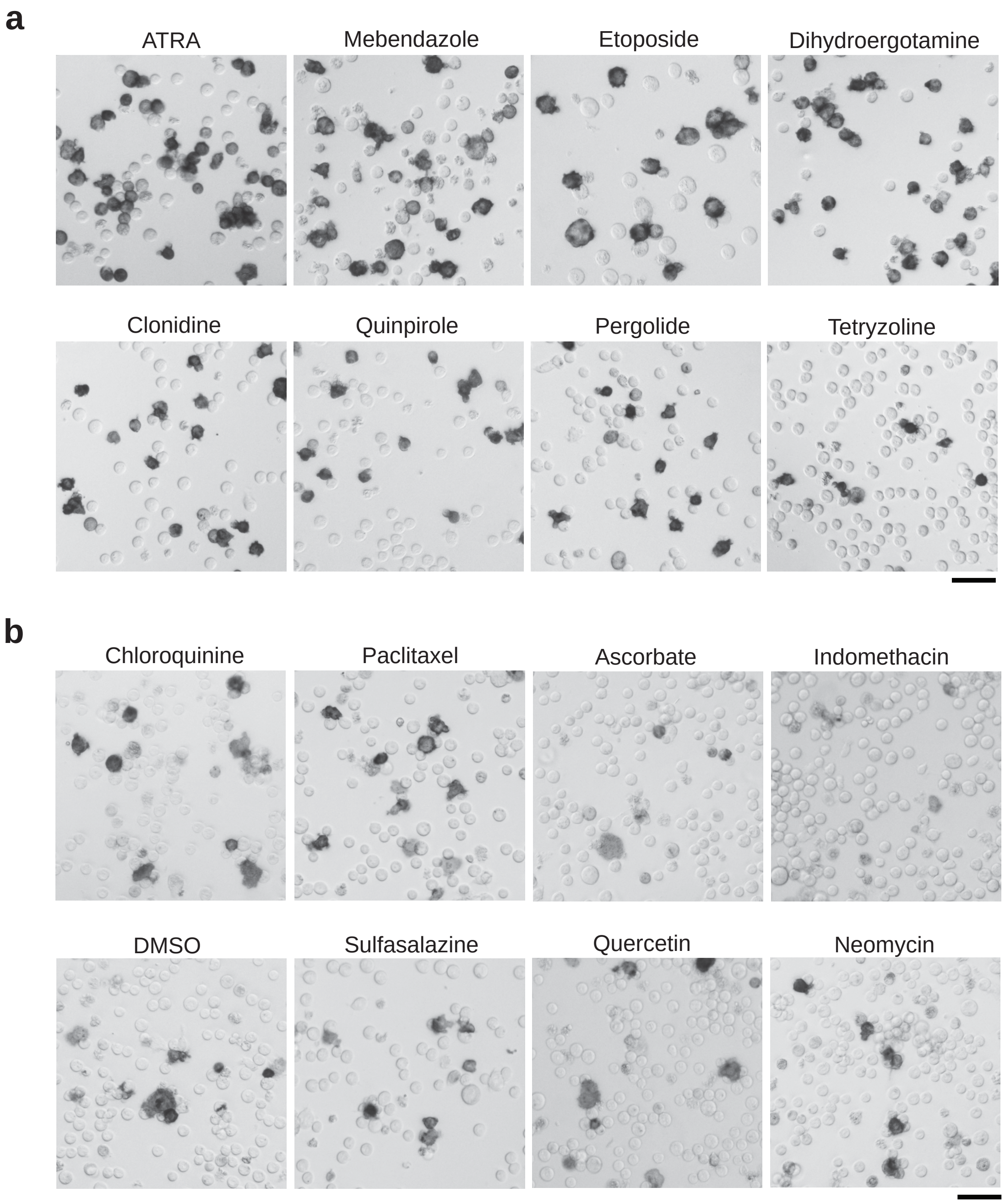

NBT staining of HL-60 leukemia cells treated with C-MAP drugs. (a) Treatments with drugs that have high LMI increases. (b) Treatments with drugs that are randomly chosen from the C-MAP drug library. ATRA, etoposide, and mebendazole were used at 1uM. Paclitaxel was used at 0.1uM. All other drugs were used at 10uM. Scale bar is 50 um.

Supplementary Fig S3

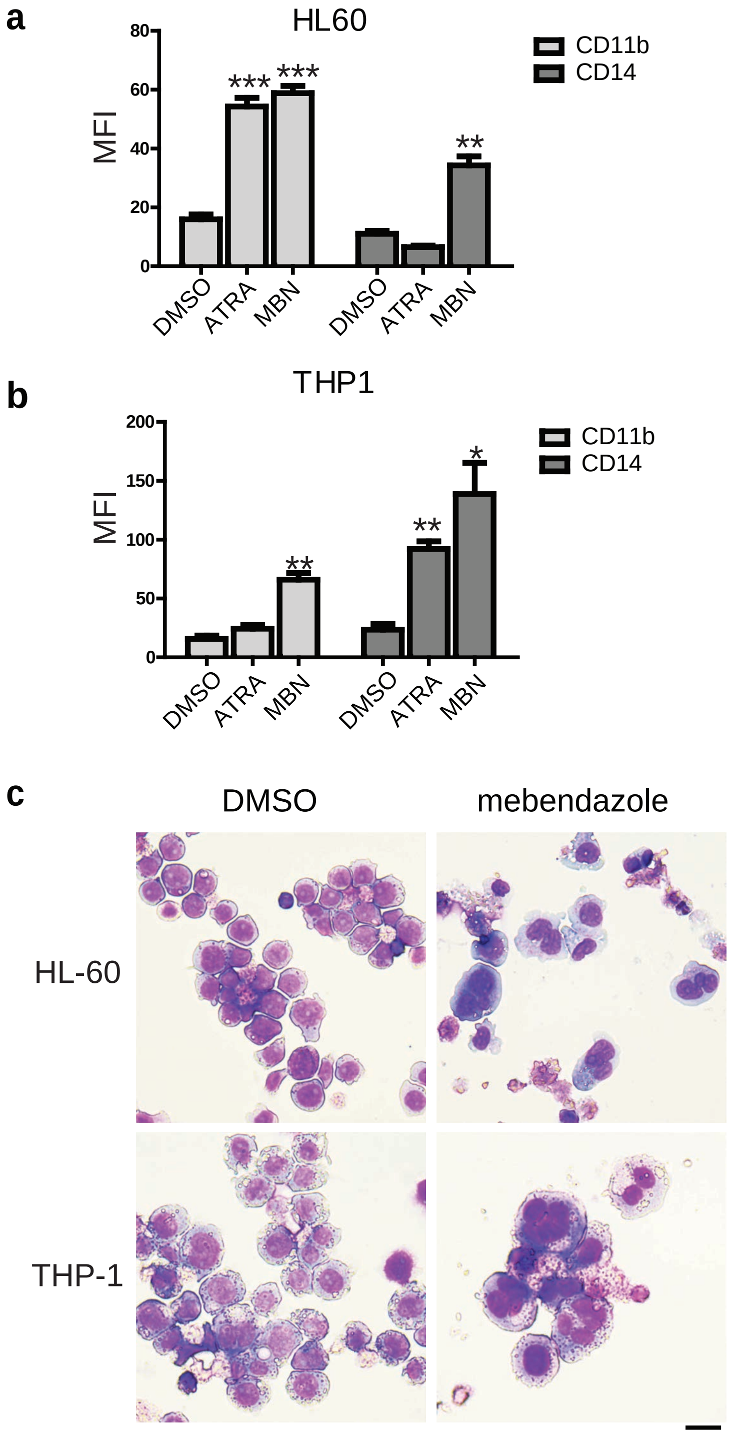

Differentiation upon mebendazole treatment in HL-60 and THP-1 cells. (a-b) Mean fluorescence intensity (MFI) of CD11b and CD14 expression in HL-60 and THP-1 leukemia cells treated with ATRA (1uM) and mebendazole (MBN) (0.33uM) for four days. *, **, and *** indicate Student's t test p value of less than 0.05., 0.01, and 0.001, respectively. (c) Morphology of HL-60 and THP-1 cells treated with mebendazole as shown by Wright's staining. Scale bar is 25 um.

Supplemetary Fig S4

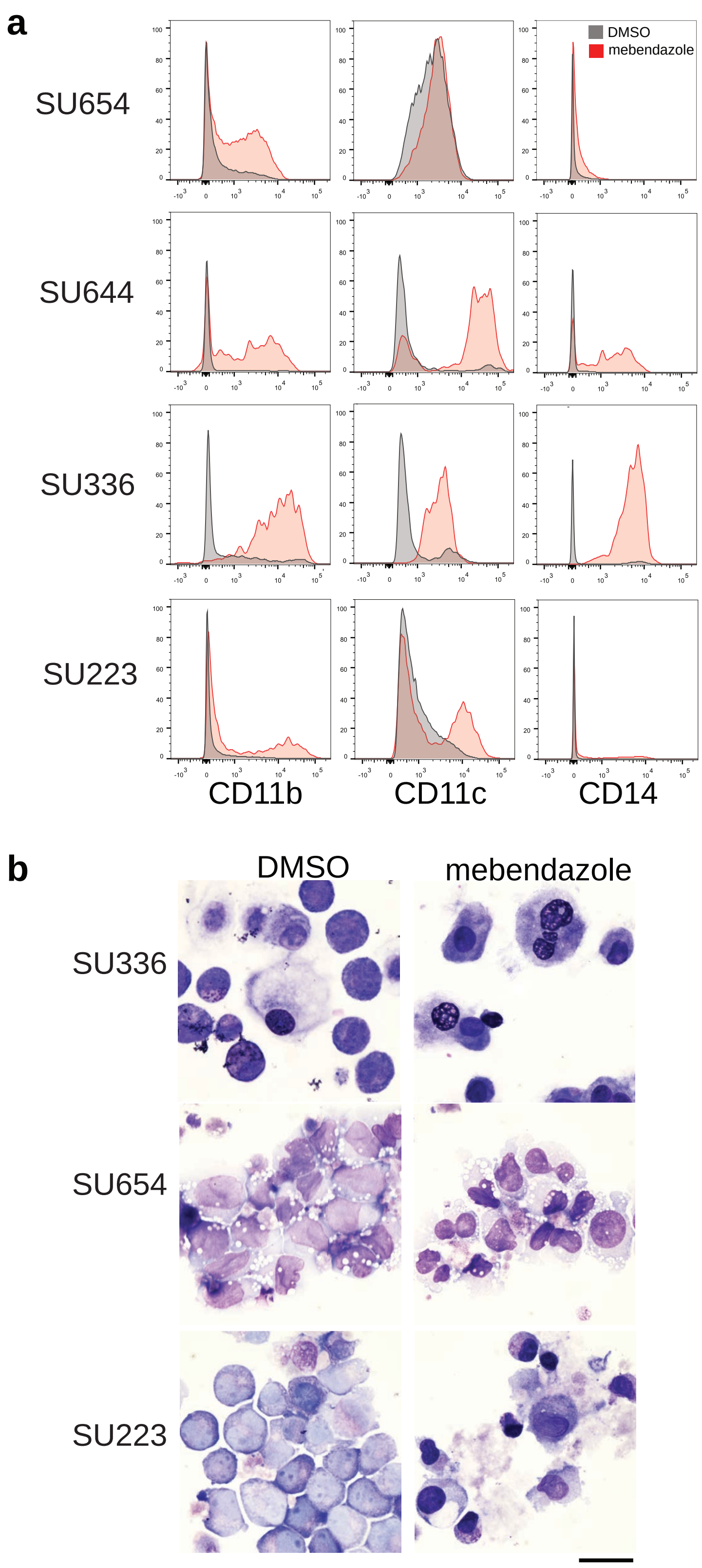

Differentiation upon mebendazole treatment in primary AML blasts from patients. (a) Flow cytometric analysis of CD11b, CD11c, and CD14 expression in primary AML samples treated with mebendazole. (b) Morphological changes of primary AML leukemic cells treated with mebendazole as shown by Wright's staining. Scale bar is 20um.

Supplementary Table S1. LMIs of the drugs from GE-HTS project.

Treatment	Array 1	Array 2	Array 3	Array 4	Array 5	Array 6	P value
Undiff	33.8	33.8	33.3	34.4	34.8	34.7	0.143
DMSO	33.6	33.9	34.5	36.5	36	35.4	1.000
Erythro	37.2	38.2	39.7				0.005
Methyl	33.6	33.4	33.5				0.072
Sulma	33.4	34	33.6				0.105
Keto	37.3	37.4	35.8				0.049
EGFR	38.6	38.1	36.7				0.009
Phen	36.4	37	39.3				0.025
5FUD	37.8	36.7	37.1				0.019
Perg	38.2	37.7	37.6				0.005
Scop	35.2	35	34.9				0.945
5FU	34.1	34.6	34.9				0.549
Caff	35.1	34.9	34.9				0.982
Apo	38.4	39.6	38.4				0.001
Cyc	36.1	36.2	35.9				0.166
VitD	46.7	47.3	47.3				0.000
PMA	40.8	39.9	40.2				0.000
ATRA	39.2	40.3	39.5				0.000

Note:
All treaments were compared to DMSO using Student's t test.
Results of the t tests were plotted in Figure 2C.
VitD, PMA and ATRA were the positive controls.
Drugs reported to have differentiation activity in the GE-HTS project were highlighted in red.
Cyc (cyclazosin) treatment did not show a statistically significant increase in LMIs.

Supplementary Table S2. LMI of 1235 arrays for the HL-60 C-MAP drug treatments

Drug Name	LMIs of Drugs	LMIs of DMSO cont	LMI Shifts	perturbation_scan_id	vehicle_scan_id4
pergolide	37.61878802	33.00109826	4.617689757	5500024024213121906560.B	.H07.G08.E09.D10.B11.A12
mebendazole	38.07285774	33.45935203	4.613505706	5500024024213121906560.G	.H01.G02.E03.D04.B05.A06
emetine	39.23596421	35.01997127	4.215992936	5.50E+25	.H01.G02.E03.D04.B05.A06
etoposide	37.04604708	33.45797002	3.58807706	622623112706.A10	.H07.G08.E09.D10.B11.A12
cephaeline	38.55816628	35.15552717	3.402639109	5500024024214122006604.G	.H01.G02.E03.D04.A06
methylergometrine	36.54416434	33.45797002	3.086194328	622623112706.D07	.H07.G08.E09.D10.B11.A12
dihydroergotamine	37.82977533	34.74789333	3.081882002	614615111406.B04	.H01.G02.E03.D04.B05.A06
tetryzoline	37.03346312	33.96076648	3.072696644	5500024025833041107269.H	.H01.G02.E03.D04.B05.A06
clonidine	36.50844166	33.4530122	3.055429454	622623112706.G06	.H01.G02.E03.B05.A06
(-)-isoprenaline	40.24499304	37.24787327	2.997119765	5500024035736031208612.C	.H01.G02.E03.D04.B05.A06
tretinoin	40.27693543	37.31290545	2.964029982	5500024035736031208612.G	.G08.E09.D10.B11.A12
quinpirole	37.07850086	34.17633596	2.9021649	5500024030760072207033.A	.H07.G08.E09.B11.A12
tretinoin	39.00594942	36.14209273	2.863856694	5500024017802120306174.G	.H01.G02.E03.D04.B05.A06
tretinoin	37.25512615	34.42118584	2.833940317	5500024030401071707292.G	.H01.G02.E03.D04.B05.A06
bromocriptine	38.21125473	35.4281341	2.783120629	612613111306.H10	.H07.G08.E09.D10.B11.A12
fenoterol	35.76007621	33.00109826	2.758977951	5500024024213121906560.G	.H07.G08.E09.D10.B11.A12
isotretinoin	35.75633787	33.00109826	2.755239602	5500024024213121906560.B	.H07.G08.E09.D10.B11.A12
cicloheximide	37.15489975	34.40088765	2.754012101	5.50E+33	.H07.G08.E09.D10.B11
podophyllotoxin	36.59543193	33.96076648	2.634665452	5500024025833041107269.A	.H01.G02.E03.D04.B05.A06
co-dergocrine mesila	37.63427517	35.01997127	2.6143039	5500024024213121906559.G	.H01.G02.E03.D04.B05.A06
tretinoin	35.93951186	33.4530122	2.486499662	622623112706.H04	.H01.G02.E03.B05.A06
menadione	37.62249204	35.15552717	2.466964871	5.50E+25	.H01.G02.E03.D04.A06
alprostadil	37.12019437	34.70962788	2.410566484	5500024030760072207033.A	.H01.G02.E03.D04.B05.A06
corbadrine	36.70730928	34.40088765	2.306421632	5500024030401071707292.G	.H07.G08.E09.D10.B11
picrotoxinin	37.3181547	35.01997127	2.298183431	5500024024213121906559.B	.H01.G02.E03.D04.B05.A06
mephentermine	36.2033931	33.96129529	2.242097807	5500024025833041107269.D	.H07.E09.D10.B11.A12
salbutamol	35.68992145	33.45935203	2.230569424	5500024024213121906560.F	.H01.G02.E03.D04.B05.A06
niclosamide	37.47162574	35.2669347	2.204691037	612613111306.B04	.H01.G02.E03.D04.B05.A06
isoetarine	36.47392415	34.40088765	2.073036503	5500024030401071707292.G	.H07.G08.E09.D10.B11
medrysone	36.00088244	33.96129529	2.039587153	5500024025833041107269.H	.H07.E09.D10.B11.A12
clenbuterol	35.48204703	33.45797002	2.024077013	622623112706.C08	.H07.G08.E09.D10.B11.A12
(+)-isoprenaline	36.23280246	34.21031976	2.022482701	5500024030760072207028.D	.H07.G08.E09.D10.B11.A12
metoprolol	35.90485872	33.96076648	1.944092245	5500024025833041107269.A	.H01.G02.E03.D04.B05.A06
thioridazine	38.06162041	36.14209273	1.919527677	5500024017802120306174.C	.H01.G02.E03.D04.B05.A06
pivampicillin	36.08538825	34.17633596	1.909052296	5500024030760072207033.H	.H07.G08.E09.B11.A12
iopanoic acid	36.03645394	34.17633596	1.860117984	5500024030760072207033.D	.H07.G08.E09.B11.A12
nimodipine	39.03814807	37.24471367	1.793434395	5500024030700072107987.A	.H01.G02.E03.D04.B05.A06
colchicine	35.24285004	33.45797002	1.784880028	622623112706.F09	.H07.G08.E09.D10.B11.A12
nocodazole	36.5178268	34.74789333	1.769933477	614615111406.C05	.H01.G02.E03.D04.B05.A06
pyrvinium	35.94053312	34.17633596	1.764197167	5500024030760072207033.F	.H07.G08.E09.B11.A12
dinoprost	36.89645834	35.15552717	1.740931174	5500024024214122006604.C	.H01.G02.E03.D04.A06
levonorgestrel	35.70091065	33.96129529	1.739615358	5500024025833041107269.H	.H07.E09.D10.B11.A12
trichostatin A	36.67804214	34.94387722	1.734164918	EC2004030506AA	EC2004030502AA
Prestwick-983	39.16936879	37.46016387	1.709204926	5500024030700072107987.B	.H07.G08.E09.D10.B11.A12
ipratropium bromide	36.76906708	35.08636713	1.682699952	6.41E+22	.H07.G08.E09.D10.B11.A12
puromycin	36.82046358	35.15552717	1.664936412	5500024024214122006604.C	.H01.G02.E03.D04.A06
thioridazine	38.94278027	37.31290545	1.629874814	5500024035736031208612.C	.G08.E09.D10.B11.A12
alpha-ergocryptine	35.5751487	33.96129529	1.613853414	5500024025833041107269.C	.H07.E09.D10.B11.A12
zardaverine	36.30579708	34.70962788	1.596169204	5500024030760072207033.C	.H01.G02.E03.D04.B05.A06
orciprenaline	36.72274614	35.13162255	1.591123588	5500024024214122006604.D	.H07.G08.E09.D10.B11.A12
vigabatrin	36.7285016	35.15552717	1.57297443	5500024024214122006604.B	.H01.G02.E03.D04.A06
genistein	37.6908361	36.14209273	1.548743373	5500024017802120306174.B	.H01.G02.E03.D04.B05.A06
labetalol	34.98443078	33.4530122	1.531418579	622623112706.H06	.H01.G02.E03.B05.A06
nadide	35.44293943	33.96076648	1.482172953	5500024025833041107269.C	.H01.G02.E03.D04.B05.A06
betazole	39.08816571	37.63864853	1.449517186	5500024024213121906564.D	.H07.G08.D10.B11.A12
probucol	34.89271771	33.45797002	1.434747692	622623112706.D08	.H07.G08.E09.D10.B11.A12
epivincamine	36.5148752	35.08636713	1.428508073	640641112706.B08	.H07.G08.E09.D10.B11.A12
tinidazole	35.38709443	33.96129529	1.425799146	5500024025833041107269.C	.H07.E09.D10.B11.A12
sulfapyridine	35.3782681	33.96076648	1.417501618	5500024025833041107269.B	.H01.G02.E03.D04.B05.A06
procarbazine	35.56014959	34.17633596	1.383813638	5500024030760072207033.C	.H07.G08.E09.B11.A12
cefoperazone	34.79522354	33.45797002	1.337253525	622623112706.A11	.H07.G08.E09.D10.B11.A12
suloctidil	38.33544692	37.00786033	1.327586586	5500024024213121906563.B	.H01.G02.E03.D04.B05.A06
terbutaline	34.7771468	33.4530122	1.324134603	622623112706.A05	.H01.G02.E03.B05.A06
oxedrine	38.55338232	37.24787327	1.30550905	5500024035736031208612.B	.H01.G02.E03.D04.B05.A06
adenosine phosphate	34.74637614	33.45797002	1.288406124	622623112706.B12	.H07.G08.E09.D10.B11.A12

minocycline	34.27960567	33.00109826	1.278507406	5500024024213121906560.B(	.H07.G08.E09.D10.B11.A12
methyldopa	34.73089855	33.45797002	1.272928536	622623112706.B08	.H07.G08.E09.D10.B11.A12
tanespimycin	37.41090461	36.14209273	1.268811883	5500024017802120306174.H	.H01.G02.E03.D04.B05.A06
sodium phenylbutyrat	36.19585457	34.94387722	1.251977344	EC2004030505AA	EC2004030502AA
sirolimus	37.38065671	36.14209273	1.238563978	5500024017802120306174.H	.H01.G02.E03.D04.B05.A06
nitrendipine	38.47482725	37.24471367	1.23011358	5500024030700072107987.D	.H01.G02.E03.D04.B05.A06
tribenoside	35.39776883	34.17633596	1.221432876	5500024030760072207033.H	.H07.G08.E09.B11.A12
fenbendazole	34.66783611	33.45935203	1.20848408	5500024024213121906560.C	.H01.G02.E03.D04.B05.A06
tolazoline	36.47538332	35.2669347	1.208448618	612613111306.A01	.H01.G02.E03.D04.B05.A06
terazosin	35.1686715	33.96076648	1.207905022	5500024025833041107269.C	.H01.G02.E03.D04.B05.A06
leflunomide	35.15656113	33.96076648	1.195794652	5500024025833041107269.A(	.H01.G02.E03.D04.B05.A06
dydrogesterone	36.21255939	35.01997127	1.19258812	5500024024213121906559.C	.H01.G02.E03.D04.B05.A06
ethotoin	36.24958436	35.05833814	1.191246214	5500024024213121906559.C	.H07.G08.E09.D10.B11.A12
sirolimus	36.12983309	34.94387722	1.18595587	EC2004030504AA	EC2004030502AA
dobutamine	34.62309094	33.45797002	1.165120926	622623112706.H11	.H07.G08.E09.D10.B11.A12
thioridazine	35.57987637	34.42118584	1.158690534	5500024030401071707292.C	.H01.G02.E03.D04.B05.A06
dipivefrine	36.24446957	35.08636713	1.158102443	640641112706.H09	.H07.G08.E09.D10.B11.A12
trichostatin A	34.60433485	33.4530122	1.151322653	622623112706.F06	.H01.G02.E03.B05.A06
disulfiram	35.89892333	34.74789333	1.151030004	614615111406.G03	.H01.G02.E03.D04.B05.A06
clorgiline	34.59688955	33.45797002	1.138919535	6.23E+21	.H07.G08.E09.D10.B11.A12
helveticoside	36.18138784	35.05833814	1.123049702	5500024024213121906559.D	.H07.G08.E09.D10.B11.A12
tanespimycin	37.25859978	36.14209273	1.116507047	5500024017802120306174.D	.H01.G02.E03.D04.B05.A06
tanespimycin	37.25782303	36.14209273	1.115730305	5500024017802120306174.D	.H01.G02.E03.D04.B05.A06
sanguinarine	35.82495099	34.70962788	1.11532311	5500024030760072207033.C	.H01.G02.E03.D04.B05.A06
felodipine	36.26394616	35.15552717	1.108418993	5500024024214122006604.F(	.H01.G02.E03.D04.A06
meptazinol	35.80590567	34.70962788	1.096277791	5500024030760072207033.G	.H01.G02.E03.D04.B05.A06
kinetin	35.049997	33.96076648	1.089230518	5500024025833041107269.G	.H01.G02.E03.D04.B05.A06
monorden	35.50906946	34.42118584	1.087883627	5.50E+22	.H01.G02.E03.D04.B05.A06
alclometasone	35.04764119	33.96076648	1.086874712	5500024025833041107269.C	.H01.G02.E03.D04.B05.A06
glafenine	34.08374321	33.00109826	1.08264495	5.50E+28	.H07.G08.E09.D10.B11.A12
gossypol	36.13134996	35.05833814	1.073011817	5500024024213121906559.B	.H07.G08.E09.D10.B11.A12
clofazimine	34.49988551	33.45797002	1.041915497	622623112706.A08	.H07.G08.E09.D10.B11.A12
albendazole	34.49144571	33.4530122	1.038433508	622623112706.H03	.H01.G02.E03.B05.A06
monorden	38.35130907	37.31290545	1.038403621	5.50E+28	.G08.E09.D10.B11.A12
sulfafurazole	34.47503053	33.45797002	1.017060511	6.23E+19	.H07.G08.E09.D10.B11.A12
lycorine	36.0718233	35.05833814	1.013485162	5500024024213121906559.C	.H07.G08.E09.D10.B11.A12
monorden	37.15160021	36.14209273	1.009507481	5.50E+22	.H01.G02.E03.D04.B05.A06
haloperidol	37.14868775	36.14209273	1.006595018	5500024017802120306174.H	.H01.G02.E03.D04.B05.A06
etilefrine	35.71525263	34.70962788	1.005624753	5500024030760072207033.C	.H01.G02.E03.D04.B05.A06
tranylcypromine	36.10656156	35.10850208	0.998059486	614615111406.F10	.H07.G08.E09.D10.B11.A12
monorden	36.15922886	35.1615202	0.99770866	EC2004073018AA	EC2004073014AA
napelline	34.9445334	33.96076648	0.983766925	5.50E+26	.H01.G02.E03.D04.B05.A06
hexetidine	36.13273385	35.15552717	0.977206684	5500024024214122006604.A(	.H01.G02.E03.D04.A06
dipyridamole	36.39708991	35.4281341	0.968955814	612613111306.F09	.H07.G08.E09.D10.B11.A12
astemizole	35.71358342	34.74789333	0.965690091	614615111406.H04	.H01.G02.E03.D04.B05.A06
strophanthidin	34.91156136	33.96076648	0.950794885	5500024025833041107269.D	.H01.G02.E03.D04.B05.A06
anisomycin	37.9584653	37.00786033	0.950604969	5500024024213121906563.A(	.H01.G02.E03.D04.B05.A06
piribedil	35.12548936	34.17633596	0.949153399	5500024030760072207033.G	.H07.G08.E09.B11.A12
monensin	34.90808649	33.96129529	0.946791204	5500024025833041107269.A	.H07.E09.D10.B11.A12
atropine oxide	35.69259977	34.74789333	0.944706438	614615111406.G04	.H01.G02.E03.D04.B05.A06
flunixin	34.87564012	33.96129529	0.914344833	5500024025833041107269.G	.H07.E09.D10.B11.A12
hexestrol	34.85938791	33.96076648	0.898621437	5500024025833041107269.F(	.H01.G02.E03.D04.B05.A06
ethaverine	35.10143555	34.21031976	0.891115792	5500024030760072207028.F	.H07.G08.E09.D10.B11.A12
(+)-chelidonine	35.95320999	35.08636713	0.866842861	640641112706.A07	.H07.G08.E09.D10.B11.A12
vidarabine	35.267069	34.40088765	0.866181352	5500024030401071707292.H	.H07.G08.E09.D10.B11
propantheline bromid	34.99429429	34.13330462	0.860989674	5500024030760072207028.C	.H01.G02.D04.B05.A06
succinylsulfathiazole	35.8762855	35.01997127	0.856314231	5500024024213121906559.A(	.H01.G02.E03.D04.B05.A06
alexidine	34.81754385	33.96129529	0.856248558	5500024025833041107269.B	.H07.E09.D10.B11.A12
trichostatin A	34.80915105	33.96129529	0.847855759	5500024025833041107269.D	.H07.E09.D10.B11.A12
ethoxyquin	34.80825795	33.96129529	0.846962658	5.50E+31	.H07.E09.D10.B11.A12
alfuzosin	34.29654598	33.45797002	0.83857596	622623112706.H08	.H07.G08.E09.D10.B11.A12
alvespimycin	35.25844788	34.42118584	0.837262041	5500024030401071707292.F(	.H01.G02.E03.D04.B05.A06
mestranol	34.96869028	34.13330462	0.835385663	5500024030760072207028.D	.H01.G02.D04.B05.A06
trichlormethiazide	34.96535166	34.13330462	0.83204704	5500024030760072207028.F(	.H01.G02.D04.B05.A06
amantadine	37.67075836	36.83996258	0.830795777	5500024024213121906563.A(	.H07.G08.E09.D10.B11.A12
dihydroergocristine	35.93561975	35.11129025	0.824329501	640641112706.B06	.H01.G02.E03.D04.B05
15-delta prostaglandi	38.13549291	37.31290545	0.822587458	5500024035736031208612.C	.G08.E09.D10.B11.A12
sulfadimidine	34.77846092	33.96129529	0.81716563	5.50E+32	.H07.E09.D10.B11.A12
meclofenoxate	34.76059775	33.96129529	0.799302458	5500024025833041107269.H	.H07.E09.D10.B11.A12

prochlorperazine	38.10699232	37.31290545	0.794086867	5500024035736031208612.F(	.G08.E09.D10.B11.A12
noretynodrel	38.43104462	37.63864853	0.792396088	5500024024213121906564.C	.H07.G08.D10.B11.A12
rolipram	38.03593614	37.24471367	0.791222466	5500024030700072107987.G	.H01.G02.E03.D04.B05.A06
sirolimus	38.10019147	37.31290545	0.787286021	5.50E+31	.G08.E09.D10.B11.A12
tanespimycin	38.09902052	37.31290545	0.786115066	5500024035736031208612.D	.G08.E09.D10.B11.A12
monobenzone	34.99520176	34.21031976	0.784882007	5500024030760072207028.C	.H07.G08.E09.D10.B11.A12
seneciphylline	35.80368957	35.01997127	0.783718301	5500024024213121906559.F(	.H01.G02.E03.D04.B05.A06
geldanamycin	38.09466582	37.31290545	0.781760365	5500024035736031208612.D	.G08.E09.D10.B11.A12
hesperetin	36.19298745	35.4281341	0.764853349	612613111306.C07	.H07.G08.E09.D10.B11.A12
tanespimycin	35.1830758	34.42118584	0.761889967	5500024030401071707292.F(	.H01.G02.E03.D04.B05.A06
prednisolone	33.7563712	33.00109826	0.755272941	5500024024213121906560.D	.H07.G08.E09.D10.B11.A12
thioguanosine	37.76008378	37.00786033	0.75222345	5500024024213121906563.H	.H01.G02.E03.D04.B05.A06
myricetin	37.58854378	36.83996258	0.748581199	5500024024213121906563.C	.H07.G08.E09.D10.B11.A12
isocarboxazid	34.7088888	33.96129529	0.747593512	5500024025833041107269.D	.H07.E09.D10.B11.A12
perhexiline	33.74539706	33.00109826	0.744298801	5500024024213121906560.A(	.H07.G08.E09.D10.B11.A12
bucladesine	35.14206007	34.40088765	0.741172422	5500024030401071707292.A(	.H07.G08.E09.D10.B11
wortmannin	36.88270884	36.14209273	0.740616116	5500024017802120306174.A(	.H01.G02.E03.D04.B05.A06
ketanserin	34.19648861	33.45797002	0.738518598	622623112706.G10	.H07.G08.E09.D10.B11.A12
Prestwick-1083	34.9141842	34.17633596	0.737848248	5500024030760072207033.B	.H07.G08.E09.B11.A12
fulvestrant	36.87950806	36.14209273	0.737415327	5500024017802120306174.H	.H01.G02.E03.D04.B05.A06
riluzole	34.19118178	33.45935203	0.73182975	5500024024213121906560.H	.H01.G02.E03.D04.B05.A06
enoxacin	34.1806638	33.45797002	0.722693783	622623112706.F08	.H07.G08.E09.D10.B11.A12
epiandrosterone	35.87780523	35.15552717	0.722278068	5500024024214122006604.D	.H01.G02.E03.D04.A06
acetylsalicylic acid	36.86361931	36.14209273	0.721526586	5500024017802120306174.H	.H01.G02.E03.D04.B05.A06
pyridoxine	35.80193583	35.08636713	0.715568706	640641112706.G11	.H07.G08.E09.D10.B11.A12
arcaine	34.84653264	34.13330462	0.713228021	5500024030760072207028.C	.H01.G02.D04.B05.A06
trichostatin A	34.16855489	33.45797002	0.710584875	622623112706.C07	.H07.G08.E09.D10.B11.A12
isoflupredone	38.34652553	37.63864853	0.707877005	5500024024213121906564.A	.H07.G08.D10.B11.A12
sirolimus	36.84428851	36.14209273	0.702195783	5.50E+25	.H01.G02.E03.D04.B05.A06
tanespimycin	36.83985847	36.14209273	0.69776574	5500024017802120306174.F(	.H01.G02.E03.D04.B05.A06
amphotericin B	35.85131543	35.15552717	0.695788266	5500024024214122006604.D	.H01.G02.E03.D04.A06
nomifensine	35.43521246	34.74789333	0.687319135	614615111406.F06	.H01.G02.E03.D04.B05.A06
chlorphenamine	35.4336778	34.74789333	0.685784468	614615111406.G05	.H01.G02.E03.D04.B05.A06
genistein	37.9976951	37.31290545	0.684789649	5500024035736031208612.B(	.G08.E09.D10.B11.A12
tanespimycin	37.99422912	37.31290545	0.681323668	5500024035736031208612.D	.G08.E09.D10.B11.A12
karakoline	35.72922332	35.05833814	0.67088518	5500024024213121906559.B	.H07.G08.E09.D10.B11.A12
riboflavin	35.75443541	35.08636713	0.668068286	6.41E+19	.H07.G08.E09.D10.B11.A12
sirolimus	36.80957594	36.14209273	0.667483214	5500024017802120306174.A(	.H01.G02.E03.D04.B05.A06
tamoxifen	35.41073796	34.74789333	0.662844629	614615111406.H05	.H01.G02.E03.D04.B05.A06
pseudopelletierine	35.74918615	35.08636713	0.662819021	640641112706.D11	.H07.G08.E09.D10.B11.A12
famprofazone	35.72076023	35.05833814	0.662422086	5500024024213121906559.G	.H07.G08.E09.D10.B11.A12
syrosingopine	35.74130524	35.08636713	0.654938112	640641112706.F07	.H07.G08.E09.D10.B11.A12
clorsulon	35.75351534	35.11129025	0.642225094	640641112706.D06	.H01.G02.E03.D04.B05
thalidomide	35.74898923	35.10850208	0.640487156	614615111406.G10	.H07.G08.E09.D10.B11.A12
homochlorcyclizine	33.63769796	33.00109826	0.636599701	5500024024213121906560.F	.H07.G08.E09.D10.B11.A12
8-azaguanine	38.27285393	37.63864853	0.634205401	5500024024213121906564.H	.H07.G08.D10.B11.A12
benfluorex	37.63020609	37.00786033	0.622345761	5500024024213121906563.H	.H01.G02.E03.D04.B05.A06
prasterone	37.86655834	37.24471367	0.621844672	5500024030700072107987.B(	.H01.G02.E03.D04.B05.A06
lanatoside C	35.67985798	35.05833814	0.621519837	5500024024213121906559.D	.H07.G08.E09.D10.B11.A12
diazoxide	35.3657685	34.74789333	0.617875174	614615111406.G01	.H01.G02.E03.D04.B05.A06
genistein	35.03766741	34.42118584	0.616481571	5500024030401071707292.B(	.H01.G02.E03.D04.B05.A06
zimeldine	36.03898119	35.4281341	0.610847094	612613111306.G10	.H07.G08.E09.D10.B11.A12
practolol	34.06755525	33.45797002	0.609585229	622623112706.H09	.H07.G08.E09.D10.B11.A12
tanespimycin	37.91973731	37.31290545	0.606831858	5500024035736031208612.F	.G08.E09.D10.B11.A12
fludroxycortide	35.66496125	35.05833814	0.606623113	5.50E+28	.H07.G08.E09.D10.B11.A12
spiperone	34.05813599	33.4530122	0.605123787	622623112706.F04	.H01.G02.E03.B05.A06
nordihydroguaiaretic a	36.74215328	36.14209273	0.600060552	5.50E+27	.H01.G02.E03.D04.B05.A06
hycanthone	34.05790944	33.45797002	0.59993942	622623112706.C09	.H07.G08.E09.D10.B11.A12
geldanamycin	36.74037057	36.14209273	0.598277838	5500024017802120306174.D	.H01.G02.E03.D04.B05.A06
thiamazole	34.559231	33.96129529	0.597935713	5500024025833041107269.C	.H07.E09.D10.B11.A12
scopolamine	34.72791807	34.13330462	0.59461345	5500024030760072207028.B(	.H01.G02.D04.B05.A06
halcinonide	35.65117568	35.05833814	0.592837537	5.50E+29	.H07.G08.E09.D10.B11.A12
monocrotaline	35.6782273	35.08636713	0.591860175	640641112706.G09	.H07.G08.E09.D10.B11.A12
S-propranolol	34.76790996	34.17633596	0.591574006	5.50E+33	.H07.G08.E09.B11.A12
mepacrine	34.04252126	33.4530122	0.589509056	6.23E+13	.H01.G02.E03.B05.A06
nystatin	35.71949145	35.13162255	0.587868895	5500024024214122006604.A	.H07.G08.E09.D10.B11.A12
cloperastine	34.5486814	33.96129529	0.587386113	5500024025833041107269.G	.H07.E09.D10.B11.A12
ritodrine	37.59247434	37.00786033	0.584614011	5.50E+23	.H01.G02.E03.D04.B05.A06
tolazamide	35.71511685	35.13162255	0.5834943	5.50E+33	.H07.G08.E09.D10.B11.A12

tretinoin	36.7741938	36.19585457	0.578339229	EC2004032013AA	EC2004032009AA
naltrexone	35.32314641	34.74789333	0.575253086	614615111406.H02	.H01.G02.E03.D04.B05.A06
furosemide	34.02773041	33.4530122	0.574718209	622623112706.B06	.H01.G02.E03.B05.A06
oxytetracycline	34.02769716	33.4530122	0.574684957	622623112706.G04	.H01.G02.E03.B05.A06
isoxsuprine	35.83649986	35.2669347	0.569565158	612613111306.D02	.H01.G02.E03.D04.B05.A06
Prestwick-685	35.62753583	35.05833814	0.569197692	5.50E+33	.H07.G08.E09.D10.B11.A12
cefmetazole	34.52638719	33.96076648	0.56562071	5500024025833041107269.D	.H01.G02.E03.D04.B05.A06
pramocaine	35.62365293	35.05833814	0.565314784	5500024024213121906559.C	.H07.G08.E09.D10.B11.A12
isosorbide	35.61874537	35.05833814	0.560407233	5500024024213121906559.F	.H07.G08.E09.D10.B11.A12
isoconazole	35.30703018	34.74789333	0.559136856	614615111406.G06	.H01.G02.E03.D04.B05.A06
sulconazole	34.76884126	34.21031976	0.558521504	5500024030760072207028.F	.H07.G08.E09.D10.B11.A12
naftifine	34.7309977	34.17633596	0.554661745	5500024030760072207033.B	.H07.G08.E09.B11.A12
altizide	34.50738611	33.96076648	0.546619635	5500024025833041107269.D	.H01.G02.E03.D04.B05.A06
etiocholanolone	35.60466483	35.05833814	0.546326693	5500024024213121906559.A	.H07.G08.E09.D10.B11.A12
fendiline	33.99399733	33.4530122	0.540985126	622623112706.C03	.H01.G02.E03.B05.A06
beta-escin	35.59642648	35.05833814	0.538088336	5500024024213121906559.C	.H07.G08.E09.D10.B11.A12
scoulerine	35.6484449	35.11129025	0.537154645	640641112706.B01	.H01.G02.E03.D04.B05
hydroquinine	35.62128647	35.08636713	0.534919346	640641112706.D12	.H07.G08.E09.D10.B11.A12
benzethonium chlorid	34.49512528	33.96076648	0.534358804	5500024025833041107269.G	.H01.G02.E03.D04.B05.A06
metitepine	33.98414598	33.45797002	0.526175965	622623112706.C11	.H07.G08.E09.D10.B11.A12
alpha-estradiol	36.66821918	36.14209273	0.526126453	5500024017802120306174.G	.H01.G02.E03.D04.B05.A06
3-hydroxy-DL-kynurer	37.53389624	37.00786033	0.526035912	5500024024213121906563.A	.H01.G02.E03.D04.B05.A06
metanephrine	35.95272	35.4281341	0.524585904	612613111306.F07	.H07.G08.E09.D10.B11.A12
butamben	34.48262707	33.96076648	0.521860595	5500024025833041107269.C	.H01.G02.E03.D04.B05.A06
N6-methyladenosine	37.52815045	37.00786033	0.520290118	5500024024213121906563.G	.H01.G02.E03.D04.B05.A06
demeclocycline	34.4791273	33.96129529	0.517832007	5500024025833041107269.H	.H07.E09.D10.B11.A12
metergoline	33.97461272	33.45797002	0.516642707	6.23E+23	.H07.G08.E09.D10.B11.A12
gemfibrozil	35.61971284	35.10850208	0.511210767	614615111406.C07	.H07.G08.E09.D10.B11.A12
naringin	35.66183818	35.15552717	0.506311015	5500024024214122006604.H	.H01.G02.E03.D04.A06
zalcitabine	35.21489349	34.70962788	0.505265604	5500024030760072207033.B	.H01.G02.E03.D04.B05.A06
etacrynic acid	33.95771786	33.4530122	0.504705659	6.23E+16	.H01.G02.E03.B05.A06
bambuterol	33.95723066	33.4530122	0.504218461	622623112706.A02	.H01.G02.E03.B05.A06
arecoline	37.5117442	37.00786033	0.503883872	5500024024213121906563.A	.H01.G02.E03.D04.B05.A06
clomipramine	33.95487233	33.4530122	0.501860125	6.23E+17	.H01.G02.E03.B05.A06
fluphenazine	37.81345797	37.31290545	0.50055252	5500024035736031208612.B	.G08.E09.D10.B11.A12
estropipate	34.46070831	33.96076648	0.499941836	5500024025833041107269.H	.H01.G02.E03.D04.B05.A06
carcinine	37.33828066	36.83996258	0.498318084	5500024024213121906563.H	.H07.G08.E09.D10.B11.A12
cinchocaine	35.75253337	35.2669347	0.485598666	612613111306.G03	.H01.G02.E03.D04.B05.A06
mianserin	35.23212454	34.74789333	0.484231208	614615111406.D02	.H01.G02.E03.D04.B05.A06
atropine	35.56859761	35.08636713	0.482230481	6.41E+21	.H07.G08.E09.D10.B11.A12
piperacetazine	37.72711972	37.24787327	0.479246444	5500024035736031208612.C	.H01.G02.E03.D04.B05.A06
prochlorperazine	34.89801171	34.42118584	0.476825876	5500024030401071707292.F	.H01.G02.E03.D04.B05.A06
zaprinast	33.93112166	33.45797002	0.47315164	622623112706.D12	.H07.G08.E09.D10.B11.A12
hydrocotarnine	35.55577107	35.08636713	0.469403938	640641112706.D08	.H07.G08.E09.D10.B11.A12
nordihydroguaiaretic a	34.8899033	34.42118584	0.468717464	5.50E+27	.H01.G02.E03.D04.B05.A06
sirolimus	37.78157442	37.31290545	0.468668964	5500024035736031208612.H	.G08.E09.D10.B11.A12
cetirizine	35.59342105	35.13162255	0.461798494	5500024024214122006604.G	.H07.G08.E09.D10.B11.A12
estradiol	36.60303743	36.14209273	0.460944704	5500024017802120306174.G	.H01.G02.E03.D04.B05.A06
fluticasone	35.16707276	34.70962788	0.457444884	5500024030760072207033.C	.H01.G02.E03.D04.B05.A06
fulvestrant	37.7655889	37.31290545	0.452683445	5500024035736031208612.B	.G08.E09.D10.B11.A12
trihexyphenidyl	35.47122921	35.01997127	0.451257943	5500024024213121906559.B	.H01.G02.E03.D04.B05.A06
cyanocobalamin	37.28830596	36.83996258	0.448343384	5500024024213121906563.F	.H07.G08.E09.D10.B11.A12
clioquinol	37.69105896	37.24471367	0.446345288	5.50E+26	.H01.G02.E03.D04.B05.A06
terfenadine	35.19272003	34.74789333	0.444826699	6.15E+15	.H01.G02.E03.D04.B05.A06
triflusal	35.55483573	35.11129025	0.44354548	640641112706.G04	.H01.G02.E03.D04.B05
wortmannin	37.7522501	37.31290545	0.439344649	5500024035736031208612.A	.G08.E09.D10.B11.A12
nitrofural	35.59474325	35.15552717	0.439216087	5500024024214122006604.A	.H01.G02.E03.D04.A06
pheneticillin	34.39948686	33.96076648	0.438720379	5500024025833041107269.A	.H01.G02.E03.D04.B05.A06
fenofibrate	33.43929933	33.00109826	0.438201071	5500024024213121906560.C	.H07.G08.E09.D10.B11.A12
ranitidine	35.54536061	35.10850208	0.436858536	614615111406.H08	.H07.G08.E09.D10.B11.A12
epitiostanol	35.1444931	34.70962788	0.434865223	5500024030760072207033.D	.H01.G02.E03.D04.B05.A06
benzathine benzylper	35.14394544	34.70962788	0.434317554	5500024030760072207033.A	.H01.G02.E03.D04.B05.A06
naringenin	37.27425794	36.83996258	0.434295361	5500024024213121906563.A	.H07.G08.E09.D10.B11.A12
ramifenazone	34.39249489	33.96076648	0.431728416	5500024025833041107269.B	.H01.G02.E03.D04.B05.A06
oxybenzone	37.67621872	37.24471367	0.431505045	5500024030700072107987.C	.H01.G02.E03.D04.B05.A06
carbenoxolone	34.56310345	34.13330462	0.429798832	5500024030760072207028.B	.H01.G02.D04.B05.A06
sirolimus	37.74114553	37.31290545	0.428240077	5500024035736031208612.A	.G08.E09.D10.B11.A12
noscapine	35.51424186	35.08636713	0.427874733	640641112706.H10	.H07.G08.E09.D10.B11.A12
mometasone	35.53869827	35.11129025	0.427408016	640641112706.A01	.H01.G02.E03.D04.B05

betahistine	35.55787998	35.13162255	0.426257424	5500024024214122006604.F(	.H07.G08.E09.D10.B11.A12
fusidic acid	37.43272858	37.00786033	0.424868254	5500024024213121906563.C	.H01.G02.E03.D04.B05.A06
desipramine	33.88177664	33.45797002	0.423806625	622623112706.F07	.H07.G08.E09.D10.B11.A12
abamectin	34.37997495	33.96076648	0.419208473	5.50E+22	.H01.G02.E03.D04.B05.A06
canadine	35.43698806	35.01997127	0.417016786	5500024024213121906559.A(	.H01.G02.E03.D04.B05.A06
methotrexate	35.84507929	35.4281341	0.416945189	612613111306.B12	.H07.G08.E09.D10.B11.A12
triflupromazine	38.05548105	37.63864853	0.416832523	5500024024213121906564.D	.H07.G08.D10.B11.A12
androsterone	37.42427549	37.00786033	0.41641516	5500024024213121906563.B(	.H01.G02.E03.D04.B05.A06
gliquidone	37.87393465	37.46016387	0.413770777	5500024030700072107987.D	.H07.G08.E09.D10.B11.A12
flunarizine	33.41153801	33.00109826	0.41043975	5500024024213121906560.F(	.H07.G08.E09.D10.B11.A12
alvespimycin	36.55161251	36.14209273	0.409519782	5500024017802120306174.F(	.H01.G02.E03.D04.B05.A06
azaperone	37.6560947	37.24787327	0.408221428	5500024035736031208612.C	.H01.G02.E03.D04.B05.A06
urapidil	37.64957319	37.24471367	0.404859523	5500024030700072107987.F(	.H01.G02.E03.D04.B05.A06
cyclobenzaprine	37.24409511	36.83996258	0.404132536	5500024024213121906563.C	.H07.G08.E09.D10.B11.A12
lovastatin	35.53309492	35.13162255	0.40147237	5500024024214122006604.B(	.H07.G08.E09.D10.B11.A12
dexamethasone	35.14869933	34.74789333	0.400806005	614615111406.B02	.H01.G02.E03.D04.B05.A06
ricinine	34.35916224	33.96076648	0.39839576	5500024025833041107269.H	.H01.G02.E03.D04.B05.A06
spectinomycin	34.52704451	34.13330462	0.393739893	5500024030760072207028.H	.H01.G02.D04.B05.A06
megestrol	37.63684356	37.24471367	0.39212989	5500024030700072107987.C	.H01.G02.E03.D04.B05.A06
coralyne	37.39920972	37.00786033	0.391349389	5500024024213121906563.B(	.H01.G02.E03.D04.B05.A06
sulfametoxydiazine	34.79168156	34.40088765	0.390793916	5500024030401071707292.G	.H07.G08.E09.D10.B11
thioridazine	35.65600009	35.2669347	0.389065388	612613111306.D03	.H01.G02.E03.D04.B05.A06
nicardipine	33.84534335	33.45797002	0.38737333	622623112706.F11	.H07.G08.E09.D10.B11.A12
digitoxigenin	37.22600045	36.83996258	0.386037867	5500024024213121906563.B(	.H07.G08.E09.D10.B11.A12
estradiol	34.80676894	34.42118584	0.385583108	5500024030401071707292.G	.H01.G02.E03.D04.B05.A06
tetrandrine	34.34456867	33.96076648	0.383802192	5.50E+23	.H01.G02.E03.D04.B05.A06
Prestwick-559	35.49491423	35.11129025	0.383623982	6.41E+15	.H01.G02.E03.D04.B05
prochlorperazine	36.52387802	36.14209273	0.381785296	5500024017802120306174.F(	.H01.G02.E03.D04.B05.A06
cefazolin	34.34028334	33.96129529	0.378988049	5500024025833041107269.D	.H07.E09.D10.B11.A12
amiloride	35.64588671	35.2669347	0.378952002	612613111306.G04	.H01.G02.E03.D04.B05.A06
beclometasone	34.51019659	34.13330462	0.376891971	5.50E+25	.H01.G02.D04.B05.A06
etanidazole	34.33732281	33.96076648	0.376556332	5500024025833041107269.G	.H01.G02.E03.D04.B05.A06
gallamine triethiodide	35.12410601	34.74789333	0.376212682	614615111406.F03	.H01.G02.E03.D04.B05.A06
etynodiol	37.61946375	37.24471367	0.374750083	5500024030700072107987.A(	.H01.G02.E03.D04.B05.A06
10-methoxyharmalan	35.48602664	35.11129025	0.374736386	640641112706.B03	.H01.G02.E03.D04.B05
flavoxate	33.37364786	33.00109826	0.3725496	5500024024213121906560.H	.H07.G08.E09.D10.B11.A12
artemisinin	35.48347351	35.11129025	0.372183256	640641112706.H06	.H01.G02.E03.D04.B05
calycanthine	35.45791681	35.08636713	0.371549687	640641112706.D07	.H07.G08.E09.D10.B11.A12
troleandomycin	35.63773148	35.2669347	0.370796773	612613111306.H04	.H01.G02.E03.D04.B05.A06
lithocholic acid	34.33155219	33.96129529	0.370256897	5500024025833041107269.C	.H07.E09.D10.B11.A12
doxazosin	34.58022057	34.21031976	0.369900815	5500024030760072207028.H	.H07.G08.E09.D10.B11.A12
raubasine	35.48036524	35.11129025	0.36907499	640641112706.A03	.H01.G02.E03.D04.B05
thiethylperazine	37.61605625	37.24787327	0.368182973	5500024035736031208612.B(	.H01.G02.E03.D04.B05.A06
indoprofen	34.49867569	34.13330462	0.365371075	5500024030760072207028.D	.H01.G02.D04.B05.A06
piperidolate	37.61241011	37.24787327	0.364536835	5500024035736031208612.G	.H01.G02.E03.D04.B05.A06
luteolin	34.57369658	34.21031976	0.36337682	5.50E+29	.H07.G08.E09.D10.B11.A12
benfotiamine	35.41994013	35.05833814	0.361601984	5500024024213121906559.G	.H07.G08.E09.D10.B11.A12
disopyramide	33.36267214	33.00109826	0.361573877	5500024024213121906560.A(	.H07.G08.E09.D10.B11.A12
tanespimycin	34.78233684	34.42118584	0.361151007	5500024030401071707292.D	.H01.G02.E03.D04.B05.A06
terconazole	35.49250883	35.13162255	0.36088628	5500024024214122006604.D	.H07.G08.E09.D10.B11.A12
piperine	37.19944927	36.83996258	0.359486696	5500024024213121906563.D	.H07.G08.E09.D10.B11.A12
iohexol	35.51458896	35.15552717	0.359061794	5500024024214122006604.A(	.H01.G02.E03.D04.A06
enalapril	33.35958708	33.00109826	0.358488821	5500024024213121906560.C	.H07.G08.E09.D10.B11.A12
rolitetracycline	34.56826548	34.21031976	0.357945724	5500024030760072207028.G	.H07.G08.E09.D10.B11.A12
fulvestrant	36.49998052	36.14209273	0.357887792	5500024017802120306174.B(	.H01.G02.E03.D04.B05.A06
viomycin	34.5326222	34.17633596	0.356286239	5500024030760072207033.A(	.H07.G08.E09.B11.A12
estradiol	37.66648356	37.31290545	0.35357811	5500024035736031208612.A(	.G08.E09.D10.B11.A12
neomycin	35.10142786	34.74789333	0.353534534	6.15E+17	.H01.G02.E03.D04.B05.A06
acemetacin	33.35308632	33.00109826	0.351988058	5500024024213121906560.A	.H07.G08.E09.D10.B11.A12
yohimbine	35.43822305	35.08636713	0.351855926	640641112706.F09	.H07.G08.E09.D10.B11.A12
cisapride	35.50609084	35.15552717	0.350563673	5500024024214122006604.D	.H01.G02.E03.D04.A06
tolbutamide	33.80933574	33.45935203	0.349983714	5500024024213121906560.C	.H01.G02.E03.D04.B05.A06
trifluoperazine	36.49169112	36.14209273	0.349598389	5500024017802120306174.D	.H01.G02.E03.D04.B05.A06
ethambutol	35.61518903	35.2669347	0.348254322	6.13E+15	.H01.G02.E03.D04.B05.A06
etodolac	35.45632802	35.10850208	0.347825944	614615111406.H11	.H07.G08.E09.D10.B11.A12
metixene	35.50190433	35.15552717	0.346377162	5500024024214122006604.C	.H01.G02.E03.D04.A06
bisoprolol	37.35351787	37.00786033	0.345657541	5500024024213121906563.D	.H01.G02.E03.D04.B05.A06
R-atenolol	35.47632857	35.13162255	0.344706021	5500024024214122006604.B(	.H07.G08.E09.D10.B11.A12
SR-95531	37.184414	36.83996258	0.344451421	5500024024213121906563.F(	.H07.G08.E09.D10.B11.A12

norfloxacin	35.4511669	35.10850208	0.34266482	614615111406.H10	.H07.G08.E09.D10.B11.A12
proadifen	34.74208912	34.40088765	0.341201474	5500024030401071707292.H	.H07.G08.E09.D10.B11
sulfanilamide	34.741365	34.40088765	0.340477348	5500024030401071707292.G	.H07.G08.E09.D10.B11
cefsulodin	34.47355288	34.13330462	0.340248266	5500024030760072207028.G	.H01.G02.D04.B05.A06
ribostamycin	34.73968054	34.40088765	0.338792895	5500024030401071707292.H	.H07.G08.E09.D10.B11
cefadroxil	37.1784657	36.83996258	0.338503125	5.50E+31	.H07.G08.E09.D10.B11.A12
Prestwick-689	34.29907614	33.96076648	0.338309659	5500024025833041107269.F(	.H01.G02.E03.D04.B05.A06
tanespimycin	34.75796603	34.42118584	0.336780194	5500024030401071707292.H	.H01.G02.E03.D04.B05.A06
sulfadimethoxine	34.29617986	33.96129529	0.334884574	5500024025833041107269.A(	.H07.E09.D10.B11.A12
naftopidil	35.04395602	34.70962788	0.334328139	5500024030760072207033.F(	.H01.G02.E03.D04.B05.A06
dextromethorphan	37.34207477	37.00786033	0.334214443	5.50E+25	.H01.G02.E03.D04.B05.A06
adrenosterone	37.79412535	37.46016387	0.333961477	5500024030700072107987.H	.H07.G08.E09.D10.B11.A12
trazodone	33.33454348	33.00109826	0.333445219	5500024024213121906560.G	.H07.G08.E09.D10.B11.A12
pipemidic acid	37.57556025	37.24471367	0.330846576	5500024030700072107987.C	.H01.G02.E03.D04.B05.A06
cefotiam	35.48416268	35.15552717	0.328635517	5500024024214122006604.A(	.H01.G02.E03.D04.A06
rescinnamine	35.34806787	35.01997127	0.328096602	5500024024213121906559.H	.H01.G02.E03.D04.B05.A06
cinchonidine	35.41118563	35.08636713	0.324818501	640641112706.C11	.H07.G08.E09.D10.B11.A12
spiramycin	34.28567047	33.96129529	0.324375186	5.50E+28	.H07.E09.D10.B11.A12
dilazep	33.78270225	33.45935203	0.323350218	5500024024213121906560.H	.H01.G02.E03.D04.B05.A06
vitexin	35.34099467	35.01997127	0.321023404	5500024024213121906559.C	.H01.G02.E03.D04.B05.A06
nifenazone	35.42783726	35.10850208	0.319335189	614615111406.B10	.H07.G08.E09.D10.B11.A12
troglitazone	36.4593759	36.14209273	0.317283173	5500024017802120306174.C	.H01.G02.E03.D04.B05.A06
benperidol	35.44867006	35.13162255	0.317047504	5500024024214122006604.F	.H07.G08.E09.D10.B11.A12
piretanide	37.56460247	37.24787327	0.316729197	5500024035736031208612.D	.H01.G02.E03.D04.B05.A06
difenidol	33.31614488	33.00109826	0.315046612	5500024024213121906560.H	.H07.G08.E09.D10.B11.A12
dacarbazine	35.40050081	35.08636713	0.314133681	640641112706.F08	.H07.G08.E09.D10.B11.A12
fluorocurarine	34.27424115	33.96076648	0.313474677	5.50E+25	.H01.G02.E03.D04.B05.A06
benzonatate	37.95103263	37.63864853	0.312384101	5500024024213121906564.F(	.H07.G08.D10.B11.A12
N-acetylmuramic acic	37.15137168	36.83996258	0.311409101	5500024024213121906563.D	.H07.G08.E09.D10.B11.A12
oxyphenbutazone	37.55910916	37.24787327	0.311235887	5500024035736031208612.A(	.H01.G02.E03.D04.B05.A06
nabumetone	37.770286	37.46016387	0.310122129	5500024030700072107987.H	.H07.G08.E09.D10.B11.A12
carbachol	34.52017387	34.21031976	0.309854113	5.50E+31	.H07.G08.E09.D10.B11.A12
ronidazole	37.55687728	37.24787327	0.309004008	5500024035736031208612.F(	.H01.G02.E03.D04.B05.A06
ivermectin	35.05595801	34.74789333	0.308064681	614615111406.H06	.H01.G02.E03.D04.B05.A06
dapsone	37.94633459	37.63864853	0.307686057	5500024024213121906564.B	.H07.G08.D10.B11.A12
merbromin	34.26737564	33.96129529	0.306080349	5500024025833041107269.A(	.H07.E09.D10.B11.A12
dimethadione	34.51598771	34.21031976	0.305667949	5500024030760072207028.G	.H07.G08.E09.D10.B11.A12
primaquine	37.14437926	36.83996258	0.304416682	5500024024213121906563.A(	.H07.G08.E09.D10.B11.A12
cyproheptadine	35.73157168	35.4281341	0.303437585	6.13E+18	.H07.G08.E09.D10.B11.A12
prednisone	35.5700989	35.2669347	0.303164194	612613111306.F06	.H01.G02.E03.D04.B05.A06
benzbromarone	37.30894268	37.00786033	0.301082348	5500024024213121906563.C	.H01.G02.E03.D04.B05.A06
tremorine	33.75276479	33.4530122	0.299752587	622623112706.B04	.H01.G02.E03.B05.A06
stachydrine	35.38534073	35.08636713	0.298973601	640641112706.H08	.H07.G08.E09.D10.B11.A12
iocetamic acid	34.42847179	34.13330462	0.295167174	5500024030760072207028.A(	.H01.G02.D04.B05.A06
mexiletine	33.75384478	33.45935203	0.294492756	5500024024213121906560.B(	.H01.G02.E03.D04.B05.A06
clemastine	33.29505652	33.00109826	0.293958252	5500024024213121906560.A	.H07.G08.E09.D10.B11.A12
vinburnine	35.37916753	35.08636713	0.2928004	640641112706.A09	.H07.G08.E09.D10.B11.A12
cefapirin	34.69263782	34.40088765	0.291750168	5500024030401071707292.C	.H07.G08.E09.D10.B11
levothyroxine sodium	37.13077467	36.83996258	0.290812094	5500024024213121906563.G	.H07.G08.E09.D10.B11.A12
streptozocin	34.25073402	33.96076648	0.289967542	5500024025833041107269.B(	.H01.G02.E03.D04.B05.A06
tetrahydroalstonine	35.37616358	35.08636713	0.289796454	640641112706.G07	.H07.G08.E09.D10.B11.A12
acebutolol	35.55627874	35.2669347	0.289344032	612613111306.C05	.H01.G02.E03.D04.B05.A06
trichostatin A	33.2901582	33.00109826	0.289059935	5500024024213121906560.H	.H07.G08.E09.D10.B11.A12
hymecromone	34.49916319	34.21031976	0.28884343	5500024030760072207028.D	.H07.G08.E09.D10.B11.A12
estradiol	34.70641817	34.42118584	0.28523233	5500024030401071707292.A(	.H01.G02.E03.D04.B05.A06
glimepiride	35.30515732	35.01997127	0.285186054	5500024024213121906559.C	.H01.G02.E03.D04.B05.A06
metrizamide	37.12421106	36.83996258	0.284248484	5500024024213121906563.F	.H07.G08.E09.D10.B11.A12
glipizide	35.71139785	35.4281341	0.283263754	612613111306.H11	.H07.G08.E09.D10.B11.A12
trichostatin A	36.42498967	36.14209273	0.282896942	5500024017802120306174.G	.H01.G02.E03.D04.B05.A06
chenodeoxycholic aci	33.28355158	33.00109826	0.28245332	5500024024213121906560.C	.H07.G08.E09.D10.B11.A12
griseofulvin	33.74166329	33.45935203	0.282311263	5500024024213121906560.H	.H01.G02.E03.D04.B05.A06
trifluoperazine	34.70019429	34.42118584	0.279008451	5500024030401071707292.D	.H01.G02.E03.D04.B05.A06
ganciclovir	34.48913801	34.21031976	0.278818256	5500024030760072207028.G	.H07.G08.E09.D10.B11.A12
cyclopenthiazide	34.98818433	34.70962788	0.27855645	5500024030760072207033.H	.H01.G02.E03.D04.B05.A06
imipramine	37.91595489	37.63864853	0.277306365	5.50E+28	.H07.G08.D10.B11.A12
praziquantel	33.73004652	33.4530122	0.277034314	622623112706.C02	.H01.G02.E03.B05.A06
benzylpenicillin	37.52433834	37.24787327	0.276465063	5500024035736031208612.B(	.H01.G02.E03.D04.B05.A06
proguanil	34.45207926	34.17633596	0.275743307	5500024030760072207033.H	.H07.G08.E09.B11.A12
mephenytoin	37.52191089	37.24787327	0.274037613	5500024035736031208612.A(	.H01.G02.E03.D04.B05.A06

quipazine	35.36000288	35.08636713	0.273635757	640641112706.A10	.H07.G08.E09.D10.B11.A12
propoxycaine	37.52150503	37.24787327	0.273631759	5500024035736031208612.A(	.H01.G02.E03.D04.B05.A06
tonzonium bromide	37.51773545	37.24471367	0.273021778	5500024030700072107987.F(	.H01.G02.E03.D04.B05.A06
methylprednisolone	33.72533409	33.4530122	0.272321889	622623112706.D01	.H01.G02.E03.B05.A06
LY-294002	36.4144016	36.14209273	0.272308874	5500024017802120306174.D	.H01.G02.E03.D04.B05.A06
fluoxetine	35.42681724	35.15552717	0.271290077	5500024024214122006604.B(	.H01.G02.E03.D04.A06
galantamine	35.29110488	35.01997127	0.271133614	5500024024213121906559.H	.H01.G02.E03.D04.B05.A06
tetramisole	35.40112368	35.13162255	0.269501129	5500024024214122006604.C	.H07.G08.E09.D10.B11.A12
theobromine	34.4007191	34.13330462	0.267414488	5500024030760072207028.F(	.H01.G02.D04.B05.A06
corticosterone	37.10679447	36.83996258	0.266831894	5500024024213121906563.H	.H07.G08.E09.D10.B11.A12
tanespimycin	37.57965012	37.31290545	0.266744666	5500024035736031208612.H	.G08.E09.D10.B11.A12
(-)-atenolol	37.50965017	37.24471367	0.264936498	5500024030700072107987.H	.H01.G02.E03.D04.B05.A06
sulfamethoxypyridazi	34.22504775	33.96129529	0.263752458	5500024025833041107269.G	.H07.E09.D10.B11.A12
apomorphine	35.6908308	35.4281341	0.262696699	612613111306.H08	.H07.G08.E09.D10.B11.A12
phenindione	35.37271634	35.11129025	0.261426088	640641112706.G05	.H01.G02.E03.D04.B05
nafcillin	34.39250555	34.13330462	0.259200936	5500024030760072207028.H	.H01.G02.D04.B05.A06
ozagrel	34.43548655	34.17633596	0.259150596	5500024030760072207033.H	.H07.G08.E09.B11.A12
tiletamine	37.71830192	37.46016387	0.258138055	5500024030700072107987.B(	.H07.G08.E09.D10.B11.A12
mebhydrolin	37.09672925	36.83996258	0.256766672	5500024024213121906563.C	.H07.G08.E09.D10.B11.A12
gabexate	34.96497148	34.70962788	0.255343594	5500024030760072207033.A(	.H01.G02.E03.D04.B05.A06
repaglinide	37.50217788	37.24787327	0.254304604	5500024035736031208612.F(	.H01.G02.E03.D04.B05.A06
fluvastatin	34.4644417	34.21031976	0.254121943	5500024030760072207028.G	.H07.G08.E09.D10.B11.A12
biperiden	35.40866001	35.15552717	0.253132844	5500024024214122006604.A(	.H01.G02.E03.D04.A06
meglumine	37.49775843	37.24471367	0.253044755	5500024030700072107987.H	.H01.G02.E03.D04.B05.A06
thiocolchicoside	35.36229225	35.11129025	0.251002004	6.41E+12	.H01.G02.E03.D04.B05
nilutamide	37.49407628	37.24471367	0.249362604	5500024030700072107987.A(	.H01.G02.E03.D04.B05.A06
nifuroxazide	35.38091959	35.13162255	0.249297034	5500024024214122006604.C	.H07.G08.E09.D10.B11.A12
dorzolamide	37.49643967	37.24787327	0.248566397	5500024035736031208612.D	.H01.G02.E03.D04.B05.A06
guanabenz	35.67656073	35.4281341	0.24842663	612613111306.A10	.H07.G08.E09.D10.B11.A12
prenylamine	35.35950161	35.11129025	0.248211364	640641112706.C02	.H01.G02.E03.D04.B05
nalbuphine	34.99512733	34.74789333	0.247233999	6.15E+12	.H01.G02.E03.D04.B05.A06
nordihydroguaiaretic a	37.55996232	37.31290545	0.247056864	5.50E+33	.G08.E09.D10.B11.A12
telenzepine	33.24782114	33.00109826	0.246722879	5.50E+29	.H07.G08.E09.D10.B11.A12
sulfamerazine	35.30451131	35.05833814	0.246173167	5500024024213121906559.F	.H07.G08.E09.D10.B11.A12
sulpiride	35.51249626	35.2669347	0.245561553	612613111306.H06	.H01.G02.E03.D04.B05.A06
lactobionic acid	37.08548915	36.83996258	0.245526569	5500024024213121906563.H	.H07.G08.E09.D10.B11.A12
phenazopyridine	34.20584883	33.96076648	0.245082355	5500024025833041107269.B(	.H01.G02.E03.D04.B05.A06
LY-294002	36.38678801	36.14209273	0.244695284	5500024017802120306174.B(	.H01.G02.E03.D04.B05.A06
rifampicin	35.37556847	35.13162255	0.243945912	5500024024214122006604.D	.H07.G08.E09.D10.B11.A12
streptomycin	33.69663781	33.4530122	0.243625606	622623112706.B03	.H01.G02.E03.B05.A06
sulfadiazine	37.8817727	37.63864853	0.243124173	5.50E+32	.H07.G08.D10.B11.A12
atropine methonitrate	37.70249953	37.46016387	0.242335662	5500024030700072107987.F(	.H07.G08.E09.D10.B11.A12
salsolidin	35.37385066	35.13162255	0.242228105	5500024024214122006604.H	.H07.G08.E09.D10.B11.A12
phenelzine	33.70024745	33.45935203	0.240895423	5500024024213121906560.C	.H01.G02.E03.D04.B05.A06
cinchonine	35.26081057	35.01997127	0.2408393	5500024024213121906559.G	.H01.G02.E03.D04.B05.A06
adiphenine	37.87886409	37.63864853	0.240215561	5500024024213121906564.A	.H07.G08.D10.B11.A12
chloroquine	35.35127037	35.11129025	0.239980115	640641112706.G06	.H01.G02.E03.D04.B05
crotamiton	34.44908007	34.21031976	0.238760315	5500024030760072207028.C	.H07.G08.E09.D10.B11.A12
mebeverine	33.68595497	33.4530122	0.23294277	622623112706.B01	.H01.G02.E03.B05.A06
naproxen	37.87145279	37.63864853	0.232804264	5500024024213121906564.A(	.H07.G08.D10.B11.A12
metamizole sodium	35.28874956	35.05833814	0.230411414	5500024024213121906559.G	.H07.G08.E09.D10.B11.A12
prazosin	37.47495277	37.24471367	0.230239095	5500024030700072107987.B(	.H01.G02.E03.D04.B05.A06
idoxuridine	35.49565291	35.2669347	0.228718204	6.13E+13	.H01.G02.E03.D04.B05.A06
ticlopidine	35.49265798	35.2669347	0.225723274	612613111306.F03	.H01.G02.E03.D04.B05.A06
eticlopride	34.4359716	34.21031976	0.225651848	5500024030760072207028.B(	.H07.G08.E09.D10.B11.A12
pimethixene	33.2253343	33.00109826	0.224236038	5500024024213121906560.D	.H07.G08.E09.D10.B11.A12
rimexolone	34.4000052	34.17633596	0.223669247	5500024030760072207033.F	.H07.G08.E09.B11.A12
nortriptyline	33.22289526	33.00109826	0.221796998	5.50E+33	.H07.G08.E09.D10.B11.A12
nicotinic acid	34.43187463	34.21031976	0.221554876	5.50E+32	.H07.G08.E09.D10.B11.A12
chloropyramine	34.35440311	34.13330462	0.221098494	5500024030760072207028.C	.H01.G02.D04.B05.A06
1,4-chrysenequinone	35.30723833	35.08636713	0.220871207	640641112706.D09	.H07.G08.E09.D10.B11.A12
cyproterone	37.46836059	37.24787327	0.220487312	5500024035736031208612.H	.H01.G02.E03.D04.B05.A06
gefitinib	35.38197686	35.1615202	0.220456653	EC2004073015AA	EC2004073014AA
naphazoline	35.48724765	35.2669347	0.22031295	612613111306.H05	.H01.G02.E03.D04.B05.A06
amitriptyline	37.85645485	37.63864853	0.217806318	5500024024213121906564.B(	.H07.G08.D10.B11.A12
homatropine	37.85376647	37.63864853	0.215117938	5500024024213121906564.F	.H07.G08.D10.B11.A12
lysergol	37.05377726	36.83996258	0.213814681	5.50E+33	.H07.G08.E09.D10.B11.A12
acetylsalicylsalicylic a	34.96048962	34.74789333	0.212596294	614615111406.F05	.H01.G02.E03.D04.B05.A06
Prestwick-972	37.67202658	37.46016387	0.211862711	5500024030700072107987.C	.H07.G08.E09.D10.B11.A12

asiaticoside	34.38800817	34.17633596	0.211672216	5500024030760072207033.H	.H07.G08.E09.B11.A12
fludrocortisone	33.67087347	33.45935203	0.211521441	5500024024213121906560.A	.H01.G02.E03.D04.B05.A06
phenacetin	35.34239372	35.13162255	0.210771164	5500024024214122006604.G	.H07.G08.E09.D10.B11.A12
LY-294002	36.35282016	36.14209273	0.210727432	5500024017802120306174.F	.H01.G02.E03.D04.B05.A06
fulvestrant	34.63037571	34.42118584	0.209189872	5500024030401071707292.H	.H01.G02.E03.D04.B05.A06
flurbiprofen	37.45382797	37.24471367	0.209114301	5500024030700072107987.C	.H01.G02.E03.D04.B05.A06
zuclopenthixol	34.91866578	34.70962788	0.209037897	5500024030760072207033.B	.H01.G02.E03.D04.B05.A06
vancomycin	35.34038123	35.13162255	0.208758679	5500024024214122006604.A	.H07.G08.E09.D10.B11.A12
tanespimycin	34.6271963	34.42118584	0.206010466	5500024030401071707292.D	.H01.G02.E03.D04.B05.A06
Prestwick-920	37.66530751	37.46016387	0.205143644	5500024030700072107987.F	.H07.G08.E09.D10.B11.A12
alverine	35.31356979	35.10850208	0.205067718	614615111406.D08	.H07.G08.E09.D10.B11.A12
vanoxerine	33.66257021	33.45797002	0.204600195	622623112706.A09	.H07.G08.E09.D10.B11.A12
dosulepin	35.31541492	35.11129025	0.204124666	640641112706.H05	.H01.G02.E03.D04.B05
thiamphenicol	37.84009099	37.63864853	0.201442462	5500024024213121906564.B	.H07.G08.D10.B11.A12
cefalotin	34.16145245	33.96076648	0.20068597	5500024025833041107269.F	.H01.G02.E03.D04.B05.A06
isometheptene	37.65996372	37.46016387	0.199799847	5500024030700072107987.A	.H07.G08.E09.D10.B11.A12
penbutolol	34.37580294	34.17633596	0.199466985	5500024030760072207033.B	.H07.G08.E09.B11.A12
(-)-MK-801	37.44322108	37.24471367	0.19850741	5.50E+22	.H01.G02.E03.D04.B05.A06
cortisone	33.19911869	33.00109826	0.198020424	5500024024213121906560.F	.H07.G08.E09.D10.B11.A12
pimozide	33.65008572	33.4530122	0.197073519	6.23E+12	.H01.G02.E03.B05.A06
iproniazid	35.30446001	35.10850208	0.195957938	614615111406.A08	.H07.G08.E09.D10.B11.A12
pirlindole	37.65535527	37.46016387	0.195191401	5500024030700072107987.B	.H07.G08.E09.D10.B11.A12
pirenperone	35.3491142	35.15552717	0.193587029	5500024024214122006604.B	.H01.G02.E03.D04.A06
haloperidol	37.50456293	37.31290545	0.191657482	5500024035736031208612.A	.G08.E09.D10.B11.A12
omeprazole	35.32199018	35.13162255	0.190367624	5500024024214122006604.G	.H07.G08.E09.D10.B11.A12
chlorpromazine	37.82868968	37.63864853	0.190041152	5500024024213121906564.C	.H07.G08.D10.B11.A12
haloperidol	35.61454297	35.4281341	0.186408876	612613111306.B09	.H07.G08.E09.D10.B11.A12
doxepin	33.18694792	33.00109826	0.185849652	5500024024213121906560.F	.H07.G08.E09.D10.B11.A12
clozapine	37.49816332	37.31290545	0.185257863	5500024035736031208612.C	.G08.E09.D10.B11.A12
flumequine	35.29372231	35.10850208	0.185220233	614615111406.D12	.H07.G08.E09.D10.B11.A12
betamethasone	33.64227234	33.45797002	0.184302326	622623112706.H12	.H07.G08.E09.D10.B11.A12
caffeic acid	34.39428805	34.21031976	0.183968298	5500024030760072207028.C	.H07.G08.E09.D10.B11.A12
neostigmine bromide	35.33529062	35.15552717	0.179763457	5500024024214122006604.F	.H01.G02.E03.D04.A06
dicloxacillin	35.33444155	35.15552717	0.178914383	5500024024214122006604.D	.H01.G02.E03.D04.A06
diphemanil metilsulfa	35.44499039	35.2669347	0.178055689	612613111306.C06	.H01.G02.E03.D04.B05.A06
cefixime	37.0136016	36.83996258	0.173639025	5500024024213121906563.G	.H07.G08.E09.D10.B11.A12
trichostatin A	35.32909794	35.15552717	0.173570774	5500024024214122006604.C	.H01.G02.E03.D04.A06
ceforanide	35.32831328	35.15552717	0.172786112	5500024024214122006604.C	.H01.G02.E03.D04.A06
gelsemine	35.191971	35.01997127	0.171999731	5500024024213121906559.B	.H01.G02.E03.D04.B05.A06
ifenprodil	33.17304696	33.00109826	0.171948699	5500024024213121906560.H	.H07.G08.E09.D10.B11.A12
velnacrine	35.32743767	35.15552717	0.171910507	5500024024214122006604.G	.H01.G02.E03.D04.A06
ergocalciferol	35.32726004	35.15552717	0.171732876	5500024024214122006604.D	.H01.G02.E03.D04.A06
nimesulide	35.2797117	35.10850208	0.171209629	614615111406.D11	.H07.G08.E09.D10.B11.A12
aminophenazone	34.91892465	34.74789333	0.171031318	614615111406.F04	.H01.G02.E03.D04.B05.A06
Prestwick-692	35.19086475	35.01997127	0.170893485	5500024024213121906559.A	.H01.G02.E03.D04.B05.A06
geldanamycin	34.59165827	34.42118584	0.170472432	5500024030401071707292.D	.H01.G02.E03.D04.B05.A06
diphenylpyraline	35.22834716	35.05833814	0.170009017	5500024024213121906559.A	.H07.G08.E09.D10.B11.A12
letrozole	34.87780351	34.70962788	0.168175627	5500024030760072207033.F	.H01.G02.E03.D04.B05.A06
hemicholinium	33.62411671	33.45797002	0.166146693	622623112706.F12	.H07.G08.E09.D10.B11.A12
proxymetacaine	37.62339443	37.46016387	0.163230559	5500024030700072107987.G	.H07.G08.E09.D10.B11.A12
flunisolide	35.22076144	35.05833814	0.162423298	5500024024213121906559.H	.H07.G08.E09.D10.B11.A12
trimethoprim	33.62156699	33.45935203	0.162214957	5500024024213121906560.F	.H01.G02.E03.D04.B05.A06
guanadrel	34.12119464	33.96129529	0.159899355	5500024025833041107269.B	.H07.E09.D10.B11.A12
ribavirin	37.61922767	37.46016387	0.159063801	5500024030700072107987.A	.H07.G08.E09.D10.B11.A12
cyclopentolate	37.4063002	37.24787327	0.158426928	5500024035736031208612.F	.H01.G02.E03.D04.B05.A06
betonicine	35.21614924	35.05833814	0.157811101	5500024024213121906559.A	.H07.G08.E09.D10.B11.A12
spironolactone	34.90484882	34.74789333	0.156955489	6.15E+13	.H01.G02.E03.D04.B05.A06
trichostatin A	34.55647422	34.40088765	0.155586574	5.50E+31	.H07.G08.E09.D10.B11
trichostatin A	34.28854502	34.13330462	0.1552404	5500024030760072207028.F	.H01.G02.D04.B05.A06
valproic acid	36.29597245	36.14209273	0.153879722	5.50E+26	.H01.G02.E03.D04.B05.A06
etomidate	34.33019335	34.17633596	0.153857393	5.50E+28	.H07.G08.E09.B11.A12
diphenhydramine	37.79175257	37.63864853	0.153104043	5500024024213121906564.A	.H07.G08.D10.B11.A12
florfenicol	37.39565091	37.24471367	0.150937238	5.50E+25	.H01.G02.E03.D04.B05.A06
esculin	34.36093462	34.21031976	0.150614868	5500024030760072207028.C	.H07.G08.E09.D10.B11.A12
trifluridine	37.39711816	37.24787327	0.149244882	5500024035736031208612.F	.H01.G02.E03.D04.B05.A06
boldine	35.16812124	35.01997127	0.148149967	5500024024213121906559.D	.H01.G02.E03.D04.B05.A06
ceftazidime	35.25894552	35.11129025	0.147655272	640641112706.F02	.H01.G02.E03.D04.B05
bretylium tosilate	34.35499389	34.21031976	0.14467413	5500024030760072207028.B	.H07.G08.E09.D10.B11.A12
carbimazole	35.29987795	35.15552717	0.144350787	5.50E+22	.H01.G02.E03.D04.A06

haloperidol	34.56547052	34.42118584	0.144284679	5500024030401071707292.H	.H01.G02.E03.D04.B05.A06
calcium folinate	34.10485477	33.96129529	0.143559483	5500024025833041107269.A	.H07.E09.D10.B11.A12
methoxamine	35.27497712	35.13162255	0.143354563	5500024024214122006604.C	.H07.G08.E09.D10.B11.A12
pinacidil	33.14407107	33.00109826	0.14297281	5500024024213121906560.B	.H07.G08.E09.D10.B11.A12
benzocaine	35.16145769	35.01997127	0.141486416	5500024024213121906559.A	.H01.G02.E03.D04.B05.A06
suxibuzone	34.10130452	33.96076648	0.140538038	5500024025833041107269.H	.H01.G02.E03.D04.B05.A06
octopamine	37.60009602	37.46016387	0.139932148	5500024030700072107987.G	.H07.G08.E09.D10.B11.A12
chlorhexidine	35.56573583	35.4281341	0.13760173	6.13E+23	.H07.G08.E09.D10.B11.A12
levocabastine	34.31311256	34.17633596	0.136776599	5500024030760072207033.G	.H07.G08.E09.B11.A12
alfadolone	37.59569651	37.46016387	0.135532641	5500024030700072107987.D	.H07.G08.E09.D10.B11.A12
butoconazole	35.29027959	35.15552717	0.134752426	5500024024214122006604.G	.H01.G02.E03.D04.A06
azapropazone	37.59488945	37.46016387	0.13472558	5500024030700072107987.A	.H07.G08.E09.D10.B11.A12
metampicillin	35.2427841	35.10850208	0.134282026	614615111406.B12	.H07.G08.E09.D10.B11.A12
amoxapine	35.56222063	35.4281341	0.13408653	612613111306.G11	.H07.G08.E09.D10.B11.A12
methyldopate	34.84322702	34.70962788	0.133599139	5500024030760072207033.A	.H01.G02.E03.D04.B05.A06
triprolidine	33.13401685	33.00109826	0.132918582	5500024024213121906560.G	.H07.G08.E09.D10.B11.A12
bupivacaine	33.1339344	33.00109826	0.132836132	5500024024213121906560.B	.H07.G08.E09.D10.B11.A12
acetylsalicylic acid	34.55326048	34.42118584	0.132074641	5500024030401071707292.H	.H01.G02.E03.D04.B05.A06
ciprofibrate	37.37945946	37.24787327	0.131586188	5.50E+23	.H01.G02.E03.D04.B05.A06
budesonide	35.24233208	35.11129025	0.131041832	640641112706.G03	.H01.G02.E03.D04.B05
calcium pantothenate	36.97081228	36.83996258	0.130849701	5500024024213121906563.G	.H07.G08.E09.D10.B11.A12
tranexamic acid	34.87841711	34.74789333	0.130523782	614615111406.A03	.H01.G02.E03.D04.B05.A06
clemizole	33.58757822	33.45935203	0.128226191	5500024024213121906560.G	.H01.G02.E03.D04.B05.A06
nicergoline	34.87552111	34.74789333	0.127627781	614615111406.F02	.H01.G02.E03.D04.B05.A06
gabapentin	34.52829819	34.40088765	0.127410542	5500024030401071707292.C	.H07.G08.E09.D10.B11
remoxipride	37.58718324	37.46016387	0.12701937	5.50E+32	.H07.G08.E09.D10.B11.A12
bupropion	33.57951553	33.4530122	0.126503333	6.23E+15	.H01.G02.E03.B05.A06
cefepime	37.37425002	37.24787327	0.126376746	5500024035736031208612.A	.H01.G02.E03.D04.B05.A06
ciclopirox	35.28082212	35.15552717	0.125294949	5500024024214122006604.B	.H01.G02.E03.D04.A06
fulvestrant	34.54647456	34.42118584	0.125288728	5500024030401071707292.B	.H01.G02.E03.D04.B05.A06
valproic acid	37.43784206	37.31290545	0.12493661	5500024035736031208612.G	.G08.E09.D10.B11.A12
Prestwick-642	35.14466333	35.01997127	0.124692056	5500024024213121906559.B	.H01.G02.E03.D04.B05.A06
chloropyrazine	37.3717267	37.24787327	0.123853429	5500024035736031208612.C	.H01.G02.E03.D04.B05.A06
gliclazide	35.2349668	35.11129025	0.123676548	640641112706.F01	.H01.G02.E03.D04.B05
naproxen	34.08387316	33.96076648	0.123106683	5500024025833041107269.C	.H01.G02.E03.D04.B05.A06
haloperidol	36.26513979	36.14209273	0.123047063	5500024017802120306174.A	.H01.G02.E03.D04.B05.A06
probenecid	35.25335337	35.13162255	0.121730816	5500024024214122006604.H	.H07.G08.E09.D10.B11.A12
nalidixic acid	33.58103914	33.45935203	0.121687107	5500024024213121906560.G	.H01.G02.E03.D04.B05.A06
LY-294002	36.26327087	36.14209273	0.121178143	5500024017802120306174.B	.H01.G02.E03.D04.B05.A06
molsidomine	35.23179249	35.11129025	0.120502237	640641112706.H03	.H01.G02.E03.D04.B05
sirolimus	34.54055646	34.42118584	0.11937062	5500024030401071707292.H	.H01.G02.E03.D04.B05.A06
papaverine	35.20540841	35.08636713	0.119041286	640641112706.H12	.H07.G08.E09.D10.B11.A12
amikacin	33.57646286	33.45797002	0.118492842	622623112706.B07	.H07.G08.E09.D10.B11.A12
aminoglutethimide	33.11797238	33.00109826	0.116874119	5.50E+32	.H07.G08.E09.D10.B11.A12
7-aminocephalospora	36.95645273	36.83996258	0.116490156	5.50E+29	.H07.G08.E09.D10.B11.A12
thiamine	35.22758925	35.11129025	0.116299003	640641112706.B04	.H01.G02.E03.D04.B05
amoxicillin	37.12414874	37.00786033	0.116288412	5500024024213121906563.H	.H01.G02.E03.D04.B05.A06
diethylstilbestrol	34.07709867	33.96129529	0.115803379	5500024025833041107269.C	.H07.E09.D10.B11.A12
trichostatin A	34.07443744	33.96076648	0.113670964	5.50E+27	.H01.G02.E03.D04.B05.A06
pindolol	34.86125417	34.74789333	0.113360845	614615111406.C04	.H01.G02.E03.D04.B05.A06
dyclonine	33.11400381	33.00109826	0.112905544	5500024024213121906560.D	.H07.G08.E09.D10.B11.A12
methotrexate	33.57077372	33.45797002	0.112803706	622623112706.F10	.H07.G08.E09.D10.B11.A12
demecarium bromide	35.198891	35.08636713	0.112523868	640641112706.C12	.H07.G08.E09.D10.B11.A12
citiolone	35.16930027	35.05833814	0.110962129	5500024024213121906559.G	.H07.G08.E09.D10.B11.A12
flutamide	33.57001214	33.45935203	0.110660113	5500024024213121906560.C	.H01.G02.E03.D04.B05.A06
fluphenazine	33.56365079	33.4530122	0.110638585	622623112706.B02	.H01.G02.E03.B05.A06
ramipril	37.35704908	37.24787327	0.109175806	5500024035736031208612.C	.H01.G02.E03.D04.B05.A06
phensuximide	34.28502753	34.17633596	0.10869157	5.50E+31	.H07.G08.E09.B11.A12
fosfosal	34.24033954	34.13330462	0.107034921	5500024030760072207028.F	.H01.G02.D04.B05.A06
estriol	37.35415371	37.24787327	0.106280439	5.50E+26	.H01.G02.E03.D04.B05.A06
norethisterone	33.10687841	33.00109826	0.105780148	5500024024213121906560.F	.H07.G08.E09.D10.B11.A12
cyclic adenosine mon	34.28206625	34.17633596	0.105730294	5500024030760072207033.C	.H07.G08.E09.B11.A12
oxybuprocaine	35.37194697	35.2669347	0.105012268	612613111306.F04	.H01.G02.E03.D04.B05.A06
isopropamide iodide	34.5049957	34.40088765	0.104108049	5.50E+29	.H07.G08.E09.D10.B11
guanethidine	33.5569603	33.4530122	0.103948101	622623112706.G05	.H01.G02.E03.B05.A06
alprenolol	33.55595665	33.4530122	0.102944444	622623112706.C01	.H01.G02.E03.B05.A06
Prestwick-981	37.56091205	37.46016387	0.100748183	5.50E+33	.H07.G08.E09.D10.B11.A12
glycocholic acid	35.25595058	35.15552717	0.100423417	5500024024214122006604.B	.H01.G02.E03.D04.A06
cytisine	35.18667621	35.08636713	0.100309077	6.41E+18	.H07.G08.E09.D10.B11.A12

nizatidine	34.31061417	34.21031976	0.10029441	5500024030760072207028.D	.H07.G08.E09.D10.B11.A12
raloxifene	34.50058761	34.40088765	0.099699962	5500024030401071707292.B	.H07.G08.E09.D10.B11
clozapine	34.52086319	34.42118584	0.099677353	5500024030401071707292.C	.H01.G02.E03.D04.B05.A06
naloxone	35.52639671	35.4281341	0.098262613	612613111306.H09	.H07.G08.E09.D10.B11.A12
thioperamide	34.30844147	34.21031976	0.098121714	5500024030760072207028.C	.H07.G08.E09.D10.B11.A12
methapyrilene	33.55537315	33.45797002	0.097403138	622623112706.H10	.H07.G08.E09.D10.B11.A12
levamisole	35.20435663	35.10850208	0.095854557	614615111406.G09	.H07.G08.E09.D10.B11.A12
piracetam	35.20712406	35.11129025	0.09583381	640641112706.H02	.H01.G02.E03.D04.B05
adipiodone	37.55590979	37.46016387	0.09574592	5500024030700072107987.G	.H07.G08.E09.D10.B11.A12
mepyramine	33.54775313	33.4530122	0.094740926	622623112706.D02	.H01.G02.E03.B05.A06
bufexamac	33.09504419	33.00109826	0.093945923	5500024024213121906560.F	.H07.G08.E09.D10.B11.A12
hexamethonium brom	35.3607717	35.2669347	0.093836997	6.13E+16	.H01.G02.E03.D04.B05.A06
foliosidine	35.15156886	35.05833814	0.093230716	5500024024213121906559.B	.H07.G08.E09.D10.B11.A12
diflunisal	35.3581299	35.2669347	0.091195192	612613111306.C02	.H01.G02.E03.D04.B05.A06
metyrapone	37.3344943	37.24471367	0.089780634	5500024030700072107987.H	.H01.G02.E03.D04.B05.A06
flufenamic acid	35.19827584	35.10850208	0.089773763	6.15E+18	.H07.G08.E09.D10.B11.A12
hexylcaine	34.48977163	34.40088765	0.088883986	5500024030401071707292.H	.H07.G08.E09.D10.B11
mafenide	35.1957671	35.10850208	0.087265022	614615111406.A07	.H07.G08.E09.D10.B11.A12
rosiglitazone	36.2289294	36.14209273	0.086836667	5500024017802120306174.C	.H01.G02.E03.D04.B05.A06
ioversol	34.29648343	34.21031976	0.086163671	5500024030760072207028.H	.H07.G08.E09.D10.B11.A12
domperidone	37.09343714	37.00786033	0.08557681	5500024024213121906563.A	.H01.G02.E03.D04.B05.A06
cefoxitin	34.4862331	34.40088765	0.085345453	5500024030401071707292.B	.H07.G08.E09.D10.B11
chlorpropamide	33.54325183	33.45797002	0.085281812	622623112706.G11	.H07.G08.E09.D10.B11.A12
levobunolol	34.21799585	34.13330462	0.084691236	5500024030760072207028.B	.H01.G02.D04.B05.A06
hecogenin	34.48272339	34.40088765	0.081835739	5500024030401071707292.F	.H07.G08.E09.D10.B11
reserpine	34.21511046	34.13330462	0.081805839	5.50E+27	.H01.G02.D04.B05.A06
4-hydroxyphenazone	35.34601754	35.2669347	0.079082838	612613111306.B03	.H01.G02.E03.D04.B05.A06
xamoterol	34.28917651	34.21031976	0.078856756	5500024030760072207028.A	.H07.G08.E09.D10.B11.A12
clozapine	37.08636984	37.00786033	0.078509508	5500024024213121906563.C	.H01.G02.E03.D04.B05.A06
acetazolamide	37.7164076	37.63864853	0.077759067	5.50E+29	.H07.G08.D10.B11.A12
cotinine	35.5053473	35.4281341	0.077213199	612613111306.G09	.H07.G08.E09.D10.B11.A12
oxymetazoline	35.18318199	35.10850208	0.074679917	614615111406.C08	.H07.G08.E09.D10.B11.A12
harmaline	35.09339326	35.01997127	0.073421994	5500024024213121906559.D	.H01.G02.E03.D04.B05.A06
acenocoumarol	34.82067277	34.74789333	0.072779438	614615111406.C06	.H01.G02.E03.D04.B05.A06
carteolol	36.9097886	36.83996258	0.06982602	5500024024213121906563.B	.H07.G08.E09.D10.B11.A12
vinpocetine	33.52274826	33.4530122	0.069736059	622623112706.F02	.H01.G02.E03.B05.A06
tiaprofenic acid	35.20037754	35.13162255	0.068754985	5500024024214122006604.C	.H07.G08.E09.D10.B11.A12
isradipine	37.52832101	37.46016387	0.068157137	5500024030700072107987.D	.H07.G08.E09.D10.B11.A12
iodixanol	34.20099881	34.13330462	0.067694188	5500024030760072207028.A	.H01.G02.D04.B05.A06
tenoxicam	35.19931002	35.13162255	0.067687462	5500024024214122006604.A	.H07.G08.E09.D10.B11.A12
bumetanide	33.06875322	33.00109826	0.067654956	5500024024213121906560.A	.H07.G08.E09.D10.B11.A12
trimipramine	34.20072306	34.13330462	0.067418443	5500024030760072207028.D	.H01.G02.D04.B05.A06
alfaxalone	37.52675714	37.46016387	0.066593273	5500024030700072107987.C	.H07.G08.E09.D10.B11.A12
loxapine	35.49463248	35.4281341	0.066498386	612613111306.F08	.H07.G08.E09.D10.B11.A12
pronetalol	34.77609581	34.70962788	0.066467933	5500024030760072207033.H	.H01.G02.E03.D04.B05.A06
verapamil	35.49455638	35.4281341	0.066422279	612613111306.H12	.H07.G08.E09.D10.B11.A12
trichostatin A	33.52442624	33.45935203	0.065074213	5500024024213121906560.A	.H01.G02.E03.D04.B05.A06
retrorsine	35.08494225	35.01997127	0.064970979	5500024024213121906559.H	.H01.G02.E03.D04.B05.A06
amylocaine	35.3277281	35.2669347	0.060793392	612613111306.C03	.H01.G02.E03.D04.B05.A06
netilmicin	34.23588078	34.17633596	0.05954482	5500024030760072207033.D	.H07.G08.E09.B11.A12
sulfacetamide	37.69791428	37.63864853	0.059265756	5500024024213121906564.C	.H07.G08.D10.B11.A12
equilin	34.26929567	34.21031976	0.058975919	5500024030760072207028.F	.H07.G08.E09.D10.B11.A12
moracizine	34.23456595	34.17633596	0.05822999	5.50E+29	.H07.G08.E09.B11.A12
metoclopramide	33.5173708	33.45935203	0.058018773	5500024024213121906560.D	.H01.G02.E03.D04.B05.A06
serotonin	35.21320754	35.15552717	0.057680374	5500024024214122006604.C	.H01.G02.E03.D04.A06
butyl hydroxybenzoat	37.29992446	37.24471367	0.055210787	5500024030700072107987.H	.H01.G02.E03.D04.B05.A06
minaprine	35.3220152	35.2669347	0.055080495	612613111306.G01	.H01.G02.E03.D04.B05.A06
bezafibrate	37.06177249	37.00786033	0.053912157	5500024024213121906563.F	.H01.G02.E03.D04.B05.A06
folic acid	35.140016	35.08636713	0.053648876	640641112706.A11	.H07.G08.E09.D10.B11.A12
sisomicin	35.18359914	35.13162255	0.051976585	5500024024214122006604.C	.H07.G08.E09.D10.B11.A12
Prestwick-682	35.07189667	35.01997127	0.051925403	5500024024213121906559.A	.H01.G02.E03.D04.B05.A06
naftidrofuryl	37.05951333	37.00786033	0.051653001	5500024024213121906563.H	.H01.G02.E03.D04.B05.A06
mifepristone	33.50335078	33.4530122	0.050338579	622623112706.D03	.H01.G02.E03.B05.A06
ketorolac	37.51018283	37.46016387	0.050018964	5500024030700072107987.G	.H07.G08.E09.D10.B11.A12
clebopride	37.05174494	37.00786033	0.043884611	5500024024213121906563.C	.H01.G02.E03.D04.B05.A06
triamterene	37.6815796	37.63864853	0.042931074	5500024024213121906564.C	.H07.G08.D10.B11.A12
trifluoperazine	37.35502106	37.31290545	0.042115607	5500024035736031208612.D	.G08.E09.D10.B11.A12
hydralazine	33.49987518	33.45935203	0.040523155	5.50E+26	.H01.G02.E03.D04.B05.A06
metrifonate	37.67890647	37.63864853	0.040257942	5500024024213121906564.G	.H07.G08.D10.B11.A12

fluphenazine	36.18229365	36.14209273	0.040200916	5500024017802120306174.B(	.H01.G02.E03.D04.B05.A06
isocorydine	35.12653333	35.08636713	0.040166202	640641112706.A08	.H07.G08.E09.D10.B11.A12
myosmine	35.09823982	35.05833814	0.039901677	5500024024213121906559.B(	.H07.G08.E09.D10.B11.A12
3-acetylcoumarin	34.24949016	34.21031976	0.039170404	5.50E+33	.H07.G08.E09.D10.B11.A12
ursodeoxycholic acid	37.49922226	37.46016387	0.039058396	5500024030700072107987.H	.H07.G08.E09.D10.B11.A12
trichostatin A	35.09706913	35.05833814	0.03873099	5500024024213121906559.A	.H07.G08.E09.D10.B11.A12
metolazone	35.46472571	35.4281341	0.036591617	612613111306.G12	.H07.G08.E09.D10.B11.A12
haloperidol	34.45755013	34.42118584	0.036364298	5500024030401071707292.A(	.H01.G02.E03.D04.B05.A06
capsaicin	34.24484799	34.21031976	0.034528238	5500024030760072207028.F(	.H07.G08.E09.D10.B11.A12
estrone	37.27777993	37.24471367	0.033066254	5500024030700072107987.G	.H01.G02.E03.D04.B05.A06
fursultiamine	34.74070665	34.70962788	0.031078766	5500024030760072207033.C	.H01.G02.E03.D04.B05.A06
ondansetron	37.27739586	37.24787327	0.029522582	5500024035736031208612.B(	.H01.G02.E03.D04.B05.A06
Prestwick-674	35.08619286	35.05833814	0.027854721	5500024024213121906559.F(	.H07.G08.E09.D10.B11.A12
zidovudine	33.48362879	33.45797002	0.025658772	622623112706.G12	.H07.G08.E09.D10.B11.A12
tropine	37.27268873	37.24787327	0.024815455	5500024035736031208612.C	.H01.G02.E03.D04.B05.A06
primidone	34.23501996	34.21031976	0.024700205	5500024030760072207028.A	.H07.G08.E09.D10.B11.A12
lorglumide	37.26870657	37.24471367	0.023992904	5500024030700072107987.F(	.H01.G02.E03.D04.B05.A06
2-aminobenzenesulfo	34.23399427	34.21031976	0.023674518	5500024030760072207028.A(	.H07.G08.E09.D10.B11.A12
fluorometholone	33.98397241	33.96076648	0.023205937	5500024025833041107269.G	.H01.G02.E03.D04.B05.A06
berberine	35.10865807	35.08636713	0.022290941	640641112706.C09	.H07.G08.E09.D10.B11.A12
minoxidil	35.2892172	35.2669347	0.022282493	612613111306.B02	.H01.G02.E03.D04.B05.A06
mevalolactone	34.42315898	34.40088765	0.022271328	5500024030401071707292.F	.H07.G08.E09.D10.B11
trimethylcolchicinic ac	35.04210086	35.01997127	0.022129595	5.50E+26	.H01.G02.E03.D04.B05.A06
pepstatin	36.86185788	36.83996258	0.021895302	5500024024213121906563.D	.H07.G08.E09.D10.B11.A12
trichostatin A	34.23190965	34.21031976	0.021589897	5500024030760072207028.B(	.H07.G08.E09.D10.B11.A12
methanthelinium bron	37.2678029	37.24787327	0.019929624	5.50E+22	.H01.G02.E03.D04.B05.A06
cycloserine	37.26720045	37.24787327	0.019327171	5.50E+25	.H01.G02.E03.D04.B05.A06
maprotiline	33.47645352	33.45797002	0.018483506	622623112706.B10	.H07.G08.E09.D10.B11.A12
meclocycline	36.85802787	36.83996258	0.018065291	5500024024213121906563.B	.H07.G08.E09.D10.B11.A12
tridihexethyl	34.19222238	34.17633596	0.015886422	5500024030760072207033.D	.H07.G08.E09.B11.A12
erythromycin	35.44302583	35.4281341	0.014891735	612613111306.G07	.H07.G08.E09.D10.B11.A12
proxyphylline	37.47284953	37.46016387	0.012685659	5500024030700072107987.F(	.H07.G08.E09.D10.B11.A12
fluspirilene	37.25736033	37.24471367	0.012646662	5500024030700072107987.D	.H01.G02.E03.D04.B05.A06
pyrantel	35.11995936	35.10850208	0.01145728	614615111406.G12	.H07.G08.E09.D10.B11.A12
betulinic acid	36.85141925	36.83996258	0.011456674	5500024024213121906563.A	.H07.G08.E09.D10.B11.A12
econazole	33.01104367	33.00109826	0.009945407	5500024024213121906560.D	.H07.G08.E09.D10.B11.A12
pentolonium	33.46885093	33.45935203	0.0094989	5500024024213121906560.F(	.H01.G02.E03.D04.B05.A06
fusaric acid	36.84931595	36.83996258	0.009353375	5500024024213121906563.H	.H07.G08.E09.D10.B11.A12
biotin	35.1638073	35.15552717	0.008280134	5500024024214122006604.G	.H01.G02.E03.D04.A06
hesperidin	37.01544172	37.00786033	0.007581389	5500024024213121906563.C	.H01.G02.E03.D04.B05.A06
cefalonium	34.71659351	34.70962788	0.006965625	5.50E+27	.H01.G02.E03.D04.B05.A06
ketoprofen	33.46598399	33.45935203	0.006631958	5500024024213121906560.D	.H01.G02.E03.D04.B05.A06
pregnenolone	35.13590816	35.13162255	0.004285609	5500024024214122006604.B	.H07.G08.E09.D10.B11.A12
heptaminol	37.64242553	37.63864853	0.003777	5500024024213121906564.B(	.H07.G08.D10.B11.A12
chlortalidone	33.45621549	33.4530122	0.003203284	622623112706.A01	.H01.G02.E03.B05.A06
Prestwick-664	35.06144086	35.05833814	0.003102718	5500024024213121906559.F(	.H07.G08.E09.D10.B11.A12
edrophonium chloride	35.43018033	35.4281341	0.002046233	612613111306.F11	.H07.G08.E09.D10.B11.A12
trichostatin A	34.71069717	34.70962788	0.001069294	5500024030760072207033.H	.H01.G02.E03.D04.B05.A06
LY-294002	34.94387722	34.94387722	3.55E-14	EC2004030503AA	EC2004030502AA
propidium iodide	33.96059986	33.96076648	-0.000166614	5500024025833041107269.A(	.H01.G02.E03.D04.B05.A06
profenamine	34.20986437	34.21031976	-0.000455381	5500024030760072207028.F	.H07.G08.E09.D10.B11.A12
digoxigenin	34.20845039	34.21031976	-0.001869365	5500024030760072207028.B	.H07.G08.E09.D10.B11.A12
LY-294002	34.41803608	34.42118584	-0.003149758	5500024030401071707292.F(	.H01.G02.E03.D04.B05.A06
brinzolamide	33.4536162	33.45797002	-0.00435382	622623112706.C10	.H07.G08.E09.D10.B11.A12
finasteride	35.0536549	35.05833814	-0.004683239	5500024024213121906559.A(	.H07.G08.E09.D10.B11.A12
sirolimus	34.41625502	34.42118584	-0.004930816	5.50E+25	.H01.G02.E03.D04.B05.A06
ofloxacin	33.45438203	33.45935203	-0.004970003	5500024024213121906560.F(	.H01.G02.E03.D04.B05.A06
dexibuprofen	37.23969399	37.24471367	-0.005019679	5500024030700072107987.C	.H01.G02.E03.D04.B05.A06
etofylline	35.10324249	35.10850208	-0.005259588	614615111406.G07	.H07.G08.E09.D10.B11.A12
flecainide	33.95557119	33.96129529	-0.005724103	5500024025833041107269.F	.H07.E09.D10.B11.A12
pipenzolate bromide	34.39485568	34.40088765	-0.006031968	5.50E+28	.H07.G08.E09.D10.B11
altretamine	37.23679683	37.24471367	-0.007916842	5500024030700072107987.D	.H01.G02.E03.D04.B05.A06
buspirone	36.99934596	37.00786033	-0.008514372	5.50E+26	.H01.G02.E03.D04.B05.A06
oleandomycin	35.41934539	35.4281341	-0.008788708	612613111306.F10	.H07.G08.E09.D10.B11.A12
bacampicillin	36.83108699	36.83996258	-0.008875591	5500024024213121906563.B(	.H07.G08.E09.D10.B11.A12
isoniazid	34.73763968	34.74789333	-0.010253653	614615111406.A01	.H01.G02.E03.D04.B05.A06
yohimbic acid	35.00858615	35.01997127	-0.011385123	5.50E+27	.H01.G02.E03.D04.B05.A06
iopamidol	34.38930678	34.40088765	-0.011580873	5500024030401071707292.C	.H07.G08.E09.D10.B11
bergenin	34.38777265	34.40088765	-0.013115002	5500024030401071707292.D	.H07.G08.E09.D10.B11

nadolol	34.11942898	34.13330462	-0.01387564	5500024030760072207028.A	.H01.G02.D04.B05.A06
skimmianine	33.94654789	33.96076648	-0.014218588	5500024025833041107269.H	.H01.G02.E03.D04.B05.A06
rosiglitazone	37.29777222	37.31290545	-0.015133235	5500024035736031208612.C	.G08.E09.D10.B11.A12
tiapride	33.44420536	33.45935203	-0.015146669	5500024024213121906560.H	.H01.G02.E03.D04.B05.A06
bromopride	35.04153327	35.05833814	-0.01680487	5500024024213121906559.F	.H07.G08.E09.D10.B11.A12
kanamycin	33.4397802	33.45797002	-0.018189819	622623112706.D09	.H07.G08.E09.D10.B11.A12
procyclidine	34.1146119	34.13330462	-0.018692719	5500024030760072207028.G	.H01.G02.D04.B05.A06
haloperidol	37.29363558	37.31290545	-0.019269868	5500024035736031208612.H	.G08.E09.D10.B11.A12
methylbenzethonium	35.03894628	35.05833814	-0.019391857	5500024024213121906559.D	.H07.G08.E09.D10.B11.A12
prilocaine	33.43888513	33.45935203	-0.020466896	5500024024213121906560.D	.H01.G02.E03.D04.B05.A06
picotamide	34.72486923	34.74789333	-0.023024102	614615111406.D05	.H01.G02.E03.D04.B05.A06
tubocurarine chloride	35.08740261	35.11129025	-0.023887639	640641112706.C03	.H01.G02.E03.D04.B05
trichostatin A	34.99590333	35.01997127	-0.02406794	5500024024213121906559.F	.H01.G02.E03.D04.B05.A06
trimetazidine	35.08647252	35.11129025	-0.024817726	6.41E+13	.H01.G02.E03.D04.B05
lynestrenol	35.4029237	35.4281341	-0.025210397	612613111306.B07	.H07.G08.E09.D10.B11.A12
carbarsone	36.8146977	36.83996258	-0.025264878	5500024024213121906563.G	.H07.G08.E09.D10.B11.A12
flucloxacillin	37.43382868	37.46016387	-0.026335186	5500024030700072107987.D	.H07.G08.E09.D10.B11.A12
rilmenidine	37.43198271	37.46016387	-0.028181156	5500024030700072107987.C	.H07.G08.E09.D10.B11.A12
latamoxef	34.18058615	34.21031976	-0.029733609	5500024030760072207028.H	.H07.G08.E09.D10.B11.A12
triamcinolone	34.71772595	34.74789333	-0.03016738	614615111406.B01	.H01.G02.E03.D04.B05.A06
eldeline	35.0273913	35.05833814	-0.030946839	5500024024213121906559.H	.H07.G08.E09.D10.B11.A12
hydroxyzine	35.39649782	35.4281341	-0.031636273	6.13E+22	.H07.G08.E09.D10.B11.A12
isoxicam	37.60632137	37.63864853	-0.032327158	5500024024213121906564.C	.H07.G08.D10.B11.A12
mephenesin	33.42670707	33.45935203	-0.032644958	5500024024213121906560.F	.H01.G02.E03.D04.B05.A06
harpagoside	34.67646533	34.70962788	-0.033162552	5500024030760072207033.B	.H01.G02.E03.D04.B05.A06
terguride	37.21089761	37.24471367	-0.033816065	5.50E+23	.H01.G02.E03.D04.B05.A06
phenylpropanolamine	33.4237992	33.45797002	-0.034170818	6.23E+18	.H07.G08.E09.D10.B11.A12
perphenazine	35.39318626	35.4281341	-0.034947836	612613111306.B10	.H07.G08.E09.D10.B11.A12
bemegride	34.17535757	34.21031976	-0.034962183	5500024030760072207028.C	.H07.G08.E09.D10.B11.A12
colistin	35.09645063	35.13162255	-0.035171921	5500024024214122006604.C	.H07.G08.E09.D10.B11.A12
tetracaine	35.07438957	35.11129025	-0.036900684	640641112706.C04	.H01.G02.E03.D04.B05
SR-95639A	36.80216213	36.83996258	-0.037800448	5500024024213121906563.C	.H07.G08.E09.D10.B11.A12
Prestwick-1103	34.1373623	34.17633596	-0.038973656	5500024030760072207033.A	.H07.G08.E09.B11.A12
enilconazole	37.42092909	37.46016387	-0.039234783	5500024030700072107987.B	.H07.G08.E09.D10.B11.A12
melatonin	35.11596524	35.15552717	-0.03956193	5500024024214122006604.F	.H01.G02.E03.D04.A06
trichostatin A	37.20663367	37.24787327	-0.041239607	5500024035736031208612.D	.H01.G02.E03.D04.B05.A06
cinnarizine	33.41174337	33.4530122	-0.041268829	622623112706.F03	.H01.G02.E03.B05.A06
flumetasone	33.91983491	33.96129529	-0.041460383	5500024025833041107269.G	.H07.E09.D10.B11.A12
dicycloverine	35.22532569	35.2669347	-0.041609014	6.13E+17	.H01.G02.E03.D04.B05.A06
tobramycin	35.08951895	35.13162255	-0.042103601	5.50E+32	.H07.G08.E09.D10.B11.A12
pilocarpine	35.11319478	35.15552717	-0.04233239	5.50E+23	.H01.G02.E03.D04.A06
niflumic acid	32.95695377	33.00109826	-0.044144494	5500024024213121906560.C	.H07.G08.E09.D10.B11.A12
desoxycortone	37.20001594	37.24471367	-0.044697734	5500024030700072107987.B	.H01.G02.E03.D04.B05.A06
clozapine	36.09730531	36.14209273	-0.044787417	5500024017802120306174.C	.H01.G02.E03.D04.B05.A06
lumicolchicine	36.79510642	36.83996258	-0.044856158	5500024024213121906563.F	.H07.G08.E09.D10.B11.A12
meclozine	35.1105889	35.15552717	-0.044938268	5500024024214122006604.H	.H01.G02.E03.D04.A06
debrisoquine	33.41222845	33.45797002	-0.045741567	622623112706.G07	.H07.G08.E09.D10.B11.A12
pempidine	35.01173671	35.05833814	-0.04660143	5500024024213121906559.H	.H07.G08.E09.D10.B11.A12
hydrastinine	35.0611964	35.10850208	-0.047305676	614615111406.B07	.H07.G08.E09.D10.B11.A12
sulfinpyrazone	33.40474931	33.4530122	-0.048262892	622623112706.C05	.H01.G02.E03.B05.A06
dizocilpine	34.69936464	34.74789333	-0.048528685	614615111406.D03	.H01.G02.E03.D04.B05.A06
pyrazinamide	35.08219943	35.13162255	-0.04942312	5.50E+28	.H07.G08.E09.D10.B11.A12
cimetidine	35.21726909	35.2669347	-0.049665612	612613111306.H03	.H01.G02.E03.D04.B05.A06
cyclizine	35.06142202	35.11129025	-0.049868235	640641112706.D01	.H01.G02.E03.D04.B05
loracarbef	34.12502085	34.17633596	-0.051315111	5500024030760072207033.C	.H07.G08.E09.B11.A12
saquinavir	37.19583916	37.24787327	-0.052034119	5500024035736031208612.G	.H01.G02.E03.D04.B05.A06
sulfathiazole	35.21481057	35.2669347	-0.052124135	612613111306.H02	.H01.G02.E03.D04.B05.A06
protoveratrine A	34.9667924	35.01997127	-0.053178869	5.50E+23	.H01.G02.E03.D04.B05.A06
ioxaglic acid	34.1214087	34.17633596	-0.054927256	5500024030760072207033.C	.H07.G08.E09.B11.A12
convolamine	35.03126923	35.08636713	-0.0550979	640641112706.C10	.H07.G08.E09.D10.B11.A12
ethionamide	35.07537299	35.13162255	-0.05624956	5500024024214122006604.B	.H07.G08.E09.D10.B11.A12
lobelanidine	35.05501755	35.11129025	-0.056272699	640641112706.A02	.H01.G02.E03.D04.B05
colecalciferol	35.09900986	35.15552717	-0.056517305	5500024024214122006604.F	.H01.G02.E03.D04.A06
6-azathymine	35.07489135	35.13162255	-0.056731201	5500024024214122006604.H	.H07.G08.E09.D10.B11.A12
diperodon	33.39589964	33.4530122	-0.057112565	622623112706.C06	.H01.G02.E03.B05.A06
bicuculline	34.96244646	35.01997127	-0.057524809	5500024024213121906559.F	.H01.G02.E03.D04.B05.A06
tyloxapol	37.18340806	37.24471367	-0.061305615	5500024030700072107987.G	.H01.G02.E03.D04.B05.A06
fenoprofen	33.89992575	33.96129529	-0.061369538	5500024025833041107269.G	.H07.E09.D10.B11.A12
tracazolate	34.6461178	34.70962788	-0.063510078	5.50E+25	.H01.G02.E03.D04.B05.A06

ketotifen	33.38912828	33.4530122	-0.063883919	622623112706.A03		.H01.G02.E03.B05.A06
tetroquinone	34.06925493	34.13330462	-0.064049685		5.50E+22	.H01.G02.D04.B05.A06
chlorpromazine	36.07800368	36.14209273	-0.064089047	5500024017802120306174.F(		.H01.G02.E03.D04.B05.A06
ascorbic acid	33.39251764	33.45797002	-0.065452379	622623112706.D11		.H07.G08.E09.D10.B11.A12
testosterone	36.94219041	37.00786033	-0.065669915	5500024024213121906563.B(		.H01.G02.E03.D04.B05.A06
sulfasalazine	35.04555672	35.11129025	-0.065733528	640641112706.D03		.H01.G02.E03.D04.B05
butacaine	34.33372104	34.40088765	-0.067166606	5500024030401071707292.D		.H07.G08.E09.D10.B11
lincomycin	32.93284678	33.00109826	-0.068251483	5500024024213121906560.G		.H07.G08.E09.D10.B11.A12
indometacin	32.93260888	33.00109826	-0.068489382	5500024024213121906560.G		.H07.G08.E09.D10.B11.A12
meprylcaine	37.17930633	37.24787327	-0.068566942	5500024035736031208612.H		.H01.G02.E03.D04.B05.A06
kaempferol	37.17892291	37.24787327	-0.068950365	5500024035736031208612.B(		.H01.G02.E03.D04.B05.A06
xylazine	34.95039088	35.01997127	-0.069580391	5500024024213121906559.H		.H01.G02.E03.D04.B05.A06
dantrolene	33.38795315	33.45935203	-0.071398883	5500024024213121906560.A(		.H01.G02.E03.D04.B05.A06
trichostatin A	35.05917398	35.13162255	-0.072448574	5500024024214122006604.F(		.H07.G08.E09.D10.B11.A12
sertaconazole	37.17256885	37.24787327	-0.075304427	5500024035736031208612.G		.H01.G02.E03.D04.B05.A06
anabasine	33.88201118	33.96076648	-0.078755297	5500024025833041107269.G		.H01.G02.E03.D04.B05.A06
(+/-)-catechin	34.05073502	34.13330462	-0.082569598	5500024030760072207028.C		.H01.G02.D04.B05.A06
clidinium bromide	34.31419324	34.40088765	-0.086694412	5500024030401071707292.B(		.H07.G08.E09.D10.B11
pargyline	35.02178553	35.10850208	-0.086716544	614615111406.F11		.H07.G08.E09.D10.B11.A12
tomatidine	34.99794551	35.08636713	-0.088421614	640641112706.H11		.H07.G08.E09.D10.B11.A12
Prestwick-665	34.9692797	35.05833814	-0.089058438		5.50E+31	.H07.G08.E09.D10.B11.A12
rosiglitazone	34.33068292	34.42118584	-0.090502915	5500024030401071707292.C		.H01.G02.E03.D04.B05.A06
alpha-estradiol	37.22206246	37.31290545	-0.090842993	5500024035736031208612.G		.G08.E09.D10.B11.A12
racecadotril	34.99530435	35.08636713	-0.091062782	640641112706.B07		.H07.G08.E09.D10.B11.A12
bethanechol	34.08499864	34.17633596	-0.091337317	5500024030760072207033.B		.H07.G08.E09.B11.A12
spaglumic acid	34.08358887	34.17633596	-0.092747082	5500024030760072207033.D		.H07.G08.E09.B11.A12
scopolamine N-oxide	35.01524555	35.10850208	-0.09325653	614615111406.F08		.H07.G08.E09.D10.B11.A12
trichostatin A	34.0830534	34.17633596	-0.093282555	5500024030760072207033.G		.H07.G08.E09.B11.A12
propylthiouracil	35.03816644	35.13162255	-0.093456114	5500024024214122006604.F		.H07.G08.E09.D10.B11.A12
midecamycin	35.33394023	35.4281341	-0.094193871	612613111306.D07		.H07.G08.E09.D10.B11.A12
bephenium hydroxyna	37.1492529	37.24471367	-0.095460766	5500024030700072107987.D		.H01.G02.E03.D04.B05.A06
mesoridazine	35.01479435	35.11129025	-0.096495901	640641112706.F06		.H01.G02.E03.D04.B05
L-methionine sulfoxim	35.03489721	35.13162255	-0.096725339	5500024024214122006604.G		.H07.G08.E09.D10.B11.A12
nipecotic acid	37.35989886	37.46016387	-0.100265011		5.50E+28	.H07.G08.E09.D10.B11.A12
chlorogenic acid	36.73809708	36.83996258	-0.101865497	5500024024213121906563.A		.H07.G08.E09.D10.B11.A12
trichostatin A	34.31544814	34.42118584	-0.105737695	5500024030401071707292.G		.H01.G02.E03.D04.B05.A06
promazine	34.95211497	35.05833814	-0.106223167	5500024024213121906559.G		.H07.G08.E09.D10.B11.A12
antazoline	33.34639035	33.4530122	-0.106621852	622623112706.F01		.H01.G02.E03.B05.A06
amrinone	34.29403864	34.40088765	-0.106849012	5500024030401071707292.D		.H07.G08.E09.D10.B11
paromomycin	34.02586829	34.13330462	-0.107436326	5500024030760072207028.B(		.H01.G02.D04.B05.A06
hydrochlorothiazide	35.15907038	35.2669347	-0.107864327	612613111306.D05		.H01.G02.E03.D04.B05.A06
phenazone	35.15858827	35.2669347	-0.108346433	612613111306.C01		.H01.G02.E03.D04.B05.A06
benzydamine	33.34217576	33.4530122	-0.110836441	622623112706.G03		.H01.G02.E03.B05.A06
dropropizine	32.88978885	33.00109826	-0.111309412	5500024024213121906560.C		.H07.G08.E09.D10.B11.A12
pyrithyldione	34.28926404	34.40088765	-0.111623613	5500024030401071707292.A(		.H07.G08.E09.D10.B11
sulfabenzamide	34.90802453	35.01997127	-0.111946737	5500024024213121906559.B(		.H01.G02.E03.D04.B05.A06
thioproperazine	34.63557815	34.74789333	-0.112315178	614615111406.C02		.H01.G02.E03.D04.B05.A06
aminophylline	34.09643693	34.21031976	-0.113882823	5500024030760072207028.F(		.H07.G08.E09.D10.B11.A12
Prestwick-967	34.59485537	34.70962788	-0.114772509	5500024030760072207033.D		.H01.G02.E03.D04.B05.A06
valproic acid	34.30427396	34.42118584	-0.116911879	5500024030401071707292.F(		.H01.G02.E03.D04.B05.A06
mepenzolate bromide	34.94060932	35.05833814	-0.117728819	5500024024213121906559.H		.H07.G08.E09.D10.B11.A12
pancuronium bromide	34.59163714	34.70962788	-0.11799074	5500024030760072207033.G		.H01.G02.E03.D04.B05.A06
conessine	34.90181244	35.01997127	-0.118158833	5500024024213121906559.G		.H01.G02.E03.D04.B05.A06
3-acetamidocoumarin	34.58757838	34.70962788	-0.122049504	5500024030760072207033.A(		.H01.G02.E03.D04.B05.A06
diethylcarbamazine	32.87882631	33.00109826	-0.122271954	5500024024213121906560.D		.H07.G08.E09.D10.B11.A12
simvastatin	34.01086392	34.13330462	-0.122440701		5.50E+26	.H01.G02.D04.B05.A06
brompheniramine	36.71667842	36.83996258	-0.123284159	5500024024213121906563.C		.H07.G08.E09.D10.B11.A12
moxisylyte	37.51457586	37.63864853	-0.124072669	5500024024213121906564.F		.H07.G08.D10.B11.A12
phthalylsulfathiazole	34.08534211	34.21031976	-0.124977651	5500024030760072207028.G		.H07.G08.E09.D10.B11.A12
delsoline	33.83547083	33.96076648	-0.125295646	5500024025833041107269.F(		.H01.G02.E03.D04.B05.A06
sulfamonomethoxine	34.27436122	34.40088765	-0.126526431	5500024030401071707292.A		.H07.G08.E09.D10.B11
trimethadione	35.00410645	35.13162255	-0.127516108	5500024024214122006604.D		.H07.G08.E09.D10.B11.A12
amodiaquine	33.32507519	33.4530122	-0.127937016	622623112706.D05		.H01.G02.E03.B05.A06
bepridil	36.87976231	37.00786033	-0.128098021	5500024024213121906563.F(		.H01.G02.E03.D04.B05.A06
betulin	34.0481478	34.17633596	-0.128188161	5500024030760072207033.F(		.H07.G08.E09.B11.A12
Prestwick-857	34.00451536	34.13330462	-0.128789253	5500024030760072207028.B(		.H01.G02.D04.B05.A06
valproic acid	34.29159356	34.42118584	-0.129592273	5500024030401071707292.A(		.H01.G02.E03.D04.B05.A06
talampicillin	34.0454833	34.17633596	-0.130852651	5500024030760072207033.F(		.H07.G08.E09.B11.A12
aciclovir	35.29579398	35.4281341	-0.132340115	612613111306.A09		.H07.G08.E09.D10.B11.A12

roxithromycin	34.00053214	34.13330462	-0.13277248	5500024030760072207028.G	.H01.G02.D04.B05.A06
pralidoxime	37.11112982	37.24471367	-0.13358385	5500024030700072107987.H	.H01.G02.E03.D04.B05.A06
troglitazone	37.17754442	37.31290545	-0.135361033	5500024035736031208612.C	.G08.E09.D10.B11.A12
SC-58125	35.02595355	35.1615202	-0.135566651	EC2004073016AA	EC2004073014AA
sulfamethizole	33.82225991	33.96076648	-0.13850657	5500024025833041107269.B	.H01.G02.E03.D04.B05.A06
dimenhydrinate	32.86170981	33.00109826	-0.139388454	5500024024213121906560.C	.H07.G08.E09.D10.B11.A12
acacetin	34.91883414	35.05833814	-0.139504	5500024024213121906559.D	.H07.G08.E09.D10.B11.A12
pheniramine	35.12713499	35.2669347	-0.139799719	612613111306.C04	.H01.G02.E03.D04.B05.A06
sulmazole	34.88000673	35.01997127	-0.139964539	5500024024213121906559.C	.H01.G02.E03.D04.B05.A06
tolmetin	33.99297122	34.13330462	-0.140333399	5500024030760072207028.C	.H01.G02.D04.B05.A06
etifenin	34.99099508	35.13162255	-0.140627472	5500024024214122006604.F	.H07.G08.E09.D10.B11.A12
metacycline	34.56748089	34.70962788	-0.142146989	5500024030760072207033.H	.H01.G02.E03.D04.B05.A06
ciprofloxacin	35.2858149	35.4281341	-0.142319199	6.13E+19	.H07.G08.E09.D10.B11.A12
propofol	34.06510768	34.21031976	-0.145212072	5500024030760072207028.D	.H07.G08.E09.D10.B11.A12
selegiline	34.98361077	35.13162255	-0.148011788	5500024024214122006604.H	.H07.G08.E09.D10.B11.A12
palmatine	34.87189502	35.01997127	-0.148076253	5500024024213121906559.F	.H01.G02.E03.D04.B05.A06
fipexide	33.30459395	33.4530122	-0.148418256	622623112706.F05	.H01.G02.E03.B05.A06
trioxysalen	33.81130935	33.96076648	-0.149457126	5500024025833041107269.F	.H01.G02.E03.D04.B05.A06
estradiol	35.9921818	36.14209273	-0.149910925	5500024017802120306174.A	.H01.G02.E03.D04.B05.A06
droperidol	36.85728516	37.00786033	-0.150575167	5500024024213121906563.C	.H01.G02.E03.D04.B05.A06
molindone	34.55820532	34.70962788	-0.151422557	5.50E+22	.H01.G02.E03.D04.B05.A06
ampicillin	35.27599959	35.4281341	-0.15213451	612613111306.D12	.H07.G08.E09.D10.B11.A12
chlorcyclizine	34.9061437	35.05833814	-0.152194444	5500024024213121906559.C	.H07.G08.E09.D10.B11.A12
diclofenamide	34.0567124	34.21031976	-0.153607357	5500024030760072207028.H	.H07.G08.E09.D10.B11.A12
mefloquine	34.59395921	34.74789333	-0.153934117	614615111406.H03	.H01.G02.E03.D04.B05.A06
famotidine	35.27026367	35.4281341	-0.157870429	612613111306.D11	.H07.G08.E09.D10.B11.A12
hydrastine hydrochlor	34.95188004	35.11129025	-0.159410207	640641112706.C05	.H01.G02.E03.D04.B05
lomefloxacin	33.29557725	33.45935203	-0.163774778	5.50E+25	.H01.G02.E03.D04.B05.A06
oxetacaine	35.10311129	35.2669347	-0.163823417	612613111306.D01	.H01.G02.E03.D04.B05.A06
idazoxan	37.08052691	37.24471367	-0.164186759	5500024030700072107987.D	.H01.G02.E03.D04.B05.A06
dihydrostreptomycin	34.58161734	34.74789333	-0.166275993	614615111406.C03	.H01.G02.E03.D04.B05.A06
iobenguane	34.94493689	35.11129025	-0.16635336	6.41E+16	.H01.G02.E03.D04.B05
carbamazepine	37.47138203	37.63864853	-0.167266499	5500024024213121906564.F	.H07.G08.D10.B11.A12
ornidazole	34.94045329	35.10850208	-0.168048782	614615111406.D07	.H07.G08.E09.D10.B11.A12
Prestwick-1085	37.07882914	37.24787327	-0.169044137	5500024035736031208612.F	.H01.G02.E03.D04.B05.A06
mefexamide	34.93684519	35.10850208	-0.171656883	614615111406.B09	.H07.G08.E09.D10.B11.A12
physostigmine	34.91437446	35.08636713	-0.171992668	640641112706.C07	.H07.G08.E09.D10.B11.A12
pizotifen	37.28740369	37.46016387	-0.172760181	5500024030700072107987.C	.H07.G08.E09.D10.B11.A12
trichostatin A	37.13814641	37.31290545	-0.174759045	5500024035736031208612.G	.G08.E09.D10.B11.A12
flucytosine	37.06959475	37.24471367	-0.175118916	5500024030700072107987.G	.H01.G02.E03.D04.B05.A06
Prestwick-864	33.95782095	34.13330462	-0.175483662	5500024030760072207028.F	.H01.G02.D04.B05.A06
tiratricol	34.93062786	35.10850208	-0.177874211	614615111406.G11	.H07.G08.E09.D10.B11.A12
pirenzepine	34.56779829	34.74789333	-0.180095036	614615111406.D06	.H01.G02.E03.D04.B05.A06
LY-294002	34.23999279	34.42118584	-0.181193047	5500024030401071707292.B	.H01.G02.E03.D04.B05.A06
butirosin	33.77693419	33.96076648	-0.18383229	5500024025833041107269.F	.H01.G02.E03.D04.B05.A06
methazolamide	34.21676288	34.40088765	-0.184124772	5500024030401071707292.C	.H07.G08.E09.D10.B11
pentamidine	34.94481375	35.13162255	-0.186808806	5500024024214122006604.F	.H07.G08.E09.D10.B11.A12
articaine	37.27280565	37.46016387	-0.187358215	5500024030700072107987.B	.H07.G08.E09.D10.B11.A12
alvespimycin	37.12059947	37.31290545	-0.192305978	5500024035736031208612.F	.G08.E09.D10.B11.A12
oxantel	36.81449914	37.00786033	-0.193361191	5500024024213121906563.F	.H01.G02.E03.D04.B05.A06
ticarcillin	37.05436916	37.24787327	-0.193504119	5500024035736031208612.D	.H01.G02.E03.D04.B05.A06
pyrimethamine	35.07070664	35.2669347	-0.196228065	612613111306.F02	.H01.G02.E03.D04.B05.A06
chlorpromazine	37.11652481	37.31290545	-0.196380639	5500024035736031208612.F	.G08.E09.D10.B11.A12
tacrine	36.81067444	37.00786033	-0.197185895	5500024024213121906563.F	.H01.G02.E03.D04.B05.A06
levopropoxyphene	33.978593	34.17633596	-0.197742956	5500024030760072207033.A	.H07.G08.E09.B11.A12
1,5-isoquinolinediol	34.96314706	35.1615202	-0.198373145	EC2004073017AA	EC2004073014AA
N-acetyl-L-aspartic ac	36.64112699	36.83996258	-0.198835584	5500024024213121906563.D	.H07.G08.E09.D10.B11.A12
etamsylate	34.51071178	34.70962788	-0.198916104	5500024030760072207033.F	.H01.G02.E03.D04.B05.A06
indapamide	33.26021853	33.45935203	-0.199133498	5500024024213121906560.B	.H01.G02.E03.D04.B05.A06
fulvestrant	37.11259634	37.31290545	-0.200309113	5500024035736031208612.H	.G08.E09.D10.B11.A12
zomepirac	34.19851769	34.40088765	-0.202369955	5500024030401071707292.F	.H07.G08.E09.D10.B11
levodopa	35.06256	35.2669347	-0.204374703	612613111306.G06	.H01.G02.E03.D04.B05.A06
dehydrocholic acid	35.22232082	35.4281341	-0.205813282	6.13E+21	.H07.G08.E09.D10.B11.A12
atractyloside	33.75471287	33.96129529	-0.206582423	5500024025833041107269.B	.H07.E09.D10.B11.A12
benzthiazide	33.9260692	34.13330462	-0.207235413	5500024030760072207028.G	.H01.G02.D04.B05.A06
alimemazine	34.19338782	34.40088765	-0.207499824	5500024030401071707292.B	.H07.G08.E09.D10.B11
promethazine	37.03658839	37.24471367	-0.208125283	5500024030700072107987.B	.H01.G02.E03.D04.B05.A06
tropicamide	33.2501084	33.45935203	-0.209243629	5.50E+23	.H01.G02.E03.D04.B05.A06
dexpanthenol	37.42851792	37.63864853	-0.210130604	5500024024213121906564.F	.H07.G08.D10.B11.A12

doxylamine	35.05660704	35.2669347	-0.210327668	612613111306.F01	.H01.G02.E03.D04.B05.A06
bacitracin	37.24884459	37.46016387	-0.211319279	5500024030700072107987.H	.H07.G08.E09.D10.B11.A12
esculetin	37.24882544	37.46016387	-0.211338427	5500024030700072107987.F	.H07.G08.E09.D10.B11.A12
sirolimus	34.20969878	34.42118584	-0.211487055	5500024030401071707292.A	.H01.G02.E03.D04.B05.A06
terguride	37.0314847	37.24471367	-0.21322897	5500024030700072107987.C	.H01.G02.E03.D04.B05.A06
betaxolol	33.24326526	33.45797002	-0.214704753	622623112706.G09	.H07.G08.E09.D10.B11.A12
guaifenesin	33.74627096	33.96129529	-0.215024333	5500024025833041107269.C	.H07.E09.D10.B11.A12
khellin	35.05157545	35.2669347	-0.215359252	612613111306.A05	.H01.G02.E03.D04.B05.A06
vincamine	33.24327295	33.45935203	-0.216079075	5500024024213121906560.A	.H01.G02.E03.D04.B05.A06
resveratrol	34.89512015	35.11129025	-0.216170097	640641112706.G01	.H01.G02.E03.D04.B05
meropenem	37.03023977	37.24787327	-0.217633509	5.50E+27	.H01.G02.E03.D04.B05.A06
fluphenazine	34.20282426	34.42118584	-0.218361572	5500024030401071707292.B	.H01.G02.E03.D04.B05.A06
oxolamine	33.91467484	34.13330462	-0.218629778	5500024030760072207028.D	.H01.G02.D04.B05.A06
bisacodyl	34.93662371	35.15552717	-0.218903458	5500024024214122006604.F	.H01.G02.E03.D04.A06
prochlorperazine	36.78787887	37.00786033	-0.219981463	5500024024213121906563.D	.H01.G02.E03.D04.B05.A06
digoxin	34.9322808	35.15552717	-0.223246366	5500024024214122006604.H	.H01.G02.E03.D04.A06
etofenamate	34.48570743	34.70962788	-0.223920453	5500024030760072207033.G	.H01.G02.E03.D04.B05.A06
theophylline	33.90936263	34.13330462	-0.223941985	5500024030760072207028.H	.H01.G02.D04.B05.A06
Prestwick-675	34.83049714	35.05833814	-0.227840996	5.50E+32	.H07.G08.E09.D10.B11.A12
alpha-estradiol	34.19329469	34.42118584	-0.227891147	5500024030401071707292.G	.H01.G02.E03.D04.B05.A06
fenbufen	33.23046029	33.45935203	-0.228891741	5.50E+22	.H01.G02.E03.D04.B05.A06
piroxicam	34.87911037	35.10850208	-0.229391702	614615111406.H09	.H07.G08.E09.D10.B11.A12
progesterone	34.92611883	35.15552717	-0.229408338	5500024024214122006604.H	.H01.G02.E03.D04.A06
hydroflumethiazide	37.40568287	37.63864853	-0.232965658	5.50E+31	.H07.G08.D10.B11.A12
chlormezanone	33.22463911	33.45797002	-0.23333091	622623112706.B09	.H07.G08.E09.D10.B11.A12
pentoxyverine	36.77296958	37.00786033	-0.234890751	5500024024213121906563.G	.H01.G02.E03.D04.B05.A06
piromidic acid	33.89833615	34.13330462	-0.234968464	5500024030760072207028.F	.H01.G02.D04.B05.A06
niridazole	34.91975529	35.15552717	-0.235771879	5.50E+26	.H01.G02.E03.D04.A06
quinisocaine	34.78391123	35.01997127	-0.236060042	5500024024213121906559.D	.H01.G02.E03.D04.B05.A06
dexpropranolol	37.00784484	37.24787327	-0.240028437	5500024035736031208612.G	.H01.G02.E03.D04.B05.A06
quercetin	34.89044508	35.13162255	-0.241177472	5500024024214122006604.A	.H07.G08.E09.D10.B11.A12
cantharidin	37.00250182	37.24471367	-0.242211855	5500024030700072107987.F	.H01.G02.E03.D04.B05.A06
furaltadone	33.71809197	33.96129529	-0.243203317	5500024025833041107269.F	.H07.E09.D10.B11.A12
iopromide	34.15753803	34.40088765	-0.243349618	5500024030401071707292.A	.H07.G08.E09.D10.B11
miconazole	35.02340337	35.2669347	-0.243531334	612613111306.F05	.H01.G02.E03.D04.B05.A06
sulfachlorpyridazine	34.81262612	35.05833814	-0.245712021	5500024024213121906559.D	.H07.G08.E09.D10.B11.A12
trichostatin A	34.83798273	35.08636713	-0.248384394	640641112706.B10	.H07.G08.E09.D10.B11.A12
sulfaguanidine	35.01744272	35.2669347	-0.249491986	612613111306.B01	.H01.G02.E03.D04.B05.A06
harman	34.77035833	35.01997127	-0.249612936	5500024024213121906559.D	.H01.G02.E03.D04.B05.A06
nitrofurantoin	33.20886067	33.45935203	-0.250491358	5500024024213121906560.F	.H01.G02.E03.D04.B05.A06
aztreonam	34.85781011	35.10850208	-0.250691965	614615111406.C12	.H07.G08.E09.D10.B11.A12
gibberellic acid	34.45713904	34.70962788	-0.25248884	5500024030760072207033.G	.H01.G02.E03.D04.B05.A06
zoxazolamine	36.75509387	37.00786033	-0.252766456	5500024024213121906563.G	.H01.G02.E03.D04.B05.A06
azlocillin	34.14428487	34.40088765	-0.25660278	5500024030401071707292.D	.H07.G08.E09.D10.B11
moxonidine	34.45287065	34.70962788	-0.256757231	5500024030760072207033.D	.H01.G02.E03.D04.B05.A06
meticrane	37.37884146	37.63864853	-0.259807065	5500024024213121906564.H	.H07.G08.D10.B11.A12
etidronic acid	33.8724712	34.13330462	-0.260833419	5500024030760072207028.H	.H01.G02.D04.B05.A06
guanfacine	36.7462256	37.00786033	-0.261634733	5.50E+22	.H01.G02.E03.D04.B05.A06
trichostatin A	35.0009978	35.2669347	-0.265936908	612613111306.G05	.H01.G02.E03.D04.B05.A06
orphenadrine	33.19308114	33.45935203	-0.266270892	5500024024213121906560.C	.H01.G02.E03.D04.B05.A06
nifurtimox	34.44224034	34.70962788	-0.267387544	5500024030760072207033.G	.H01.G02.E03.D04.B05.A06
sparteine	34.75116839	35.01997127	-0.268802883	5500024024213121906559.G	.H01.G02.E03.D04.B05.A06
amiprilose	33.86250501	34.13330462	-0.27079961	5.50E+23	.H01.G02.D04.B05.A06
estradiol	36.7363699	37.00786033	-0.271490428	5500024024213121906563.B	.H01.G02.E03.D04.B05.A06
glycopyrronium bromi	33.68783247	33.96129529	-0.27346282	5500024025833041107269.D	.H07.E09.D10.B11.A12
eucatropine	33.6860598	33.96129529	-0.275235486	5500024025833041107269.F	.H07.E09.D10.B11.A12
bendroflumethiazide	33.68380822	33.96129529	-0.277487065	5500024025833041107269.F	.H07.E09.D10.B11.A12
liothyronine	33.85524931	34.13330462	-0.278055304	5500024030760072207028.H	.H01.G02.D04.B05.A06
natamycin	36.96969994	37.24787327	-0.278173332	5500024035736031208612.H	.H01.G02.E03.D04.B05.A06
aminocaproic acid	37.18195332	37.46016387	-0.27821055	5.50E+29	.H07.G08.E09.D10.B11.A12
nisoxetine	37.18140438	37.46016387	-0.278759484	5500024030700072107987.F	.H07.G08.E09.D10.B11.A12
dioxybenzone	36.9654051	37.24471367	-0.279308575	5500024030700072107987.B	.H01.G02.E03.D04.B05.A06
roxarsone	33.89411256	34.17633596	-0.282223396	5500024030760072207033.G	.H07.G08.E09.B11.A12
carisoprodol	36.55405103	36.83996258	-0.285911544	5500024024213121906563.G	.H07.G08.E09.D10.B11.A12
aminohippuric acid	36.95729557	37.24471367	-0.287418098	5500024030700072107987.F	.H01.G02.E03.D04.B05.A06
lisinopril	32.71242183	33.00109826	-0.288676436	5500024024213121906560.H	.H07.G08.E09.D10.B11.A12
metformin	37.3473092	37.63864853	-0.291339327	5500024024213121906564.D	.H07.G08.D10.B11.A12
drofenine	34.10829735	34.40088765	-0.2925903	5500024030401071707292.F	.H07.G08.E09.D10.B11
ethisterone	33.16535665	33.45935203	-0.293995377	5500024024213121906560.A	.H01.G02.E03.D04.B05.A06

oxybutynin	33.15679555	33.4530122	-0.296216648	622623112706.G01	.H01.G02.E03.B05.A06
furazolidone	33.83597628	34.13330462	-0.297328339	5500024030760072207028.A	.H01.G02.D04.B05.A06
deptropine	37.16104221	37.46016387	-0.299121661	5500024030700072107987.A	.H07.G08.E09.D10.B11.A12
trichostatin A	37.15982442	37.46016387	-0.300339444	5500024030700072107987.G	.H07.G08.E09.D10.B11.A12
loperamide	35.12205899	35.4281341	-0.306075108	612613111306.C09	.H07.G08.E09.D10.B11.A12
Prestwick-984	34.40089176	34.70962788	-0.308736123	5500024030760072207033.H	.H01.G02.E03.D04.B05.A06
propranolol	33.90035856	34.21031976	-0.309961201	5500024030760072207028.B	.H07.G08.E09.D10.B11.A12
denatonium benzoate	37.1480139	37.46016387	-0.312149964	5.50E+31	.H07.G08.E09.D10.B11.A12
solasodine	34.74483395	35.05833814	-0.313504189	5500024024213121906559.H	.H07.G08.E09.D10.B11.A12
phentolamine	33.14338365	33.45935203	-0.315968376	5500024024213121906560.B	.H01.G02.E03.D04.B05.A06
buflomedil	36.52345469	36.83996258	-0.31650789	5500024024213121906563.B	.H07.G08.E09.D10.B11.A12
clomifene	36.69070222	37.00786033	-0.31715811	5500024024213121906563.G	.H01.G02.E03.D04.B05.A06
heliotrine	34.73632539	35.05833814	-0.322012751	5500024024213121906559.F	.H07.G08.E09.D10.B11.A12
felbinac	33.88561977	34.21031976	-0.324699986	5500024030760072207028.A	.H07.G08.E09.D10.B11.A12
ciclosporin	36.51302164	36.83996258	-0.326940943	5500024024213121906563.C	.H07.G08.E09.D10.B11.A12
lymecycline	33.84880532	34.17633596	-0.327530639	5500024030760072207033.F	.H07.G08.E09.B11.A12
canavanine	34.69207838	35.01997127	-0.327892886	5500024024213121906559.F	.H01.G02.E03.D04.B05.A06
lidocaine	34.93703463	35.2669347	-0.329900073	612613111306.B06	.H01.G02.E03.D04.B05.A06
ouabain	36.67557531	37.00786033	-0.332285022	5500024024213121906563.A	.H01.G02.E03.D04.B05.A06
chloramphenicol	37.30591632	37.63864853	-0.332732213	5500024024213121906564.H	.H07.G08.D10.B11.A12
trichostatin A	36.67357514	37.00786033	-0.334285189	5500024024213121906563.D	.H01.G02.E03.D04.B05.A06
LY-294002	36.97836664	37.31290545	-0.334538813	5500024035736031208612.F	.G08.E09.D10.B11.A12
doxycycline	34.0653892	34.40088765	-0.335498445	5500024030401071707292.B	.H07.G08.E09.D10.B11
proglumide	33.12330898	33.45935203	-0.336043046	5500024024213121906560.B	.H01.G02.E03.D04.B05.A06
benzamil	34.72211854	35.05833814	-0.336219603	5500024024213121906559.B	.H07.G08.E09.D10.B11.A12
tiabendazole	34.793074	35.13162255	-0.338548553	5.50E+29	.H07.G08.E09.D10.B11.A12
kawain	33.11947988	33.45935203	-0.339872153	5500024024213121906560.G	.H01.G02.E03.D04.B05.A06
halofantrine	37.11953012	37.46016387	-0.340633745	5500024030700072107987.D	.H07.G08.E09.D10.B11.A12
epirizole	37.29636492	37.63864853	-0.342283613	5500024024213121906564.F	.H07.G08.D10.B11.A12
Chicago Sky Blue 6B	36.49611758	36.83996258	-0.343844999	5500024024213121906563.D	.H07.G08.E09.D10.B11.A12
imipenem	34.7664686	35.11129025	-0.344821649	640641112706.F05	.H01.G02.E03.D04.B05
amprolium	34.92157098	35.2669347	-0.345363724	6.13E+12	.H01.G02.E03.D04.B05.A06
acetylsalicylic acid	36.96199131	37.31290545	-0.350914141	5500024035736031208612.H	.G08.E09.D10.B11.A12
cloxacillin	34.75750575	35.10850208	-0.350996329	614615111406.A09	.H07.G08.E09.D10.B11.A12
trichostatin A	34.75440601	35.10850208	-0.354096064	6.15E+19	.H07.G08.E09.D10.B11.A12
midodrine	34.39368705	34.74789333	-0.354206279	614615111406.A05	.H01.G02.E03.D04.B05.A06
diltiazem	35.07338808	35.4281341	-0.354746013	612613111306.C08	.H07.G08.E09.D10.B11.A12
LY-294002	36.95787404	37.31290545	-0.355031407	5500024035736031208612.B	.G08.E09.D10.B11.A12
sitosterol	34.35409907	34.70962788	-0.355528814	5500024030760072207033.F	.H01.G02.E03.D04.B05.A06
trichostatin A	34.75370482	35.11129025	-0.357585432	640641112706.D02	.H01.G02.E03.D04.B05
cefotaxime	34.38400123	34.74789333	-0.363892103	614615111406.C01	.H01.G02.E03.D04.B05.A06
diloxanide	33.84486409	34.21031976	-0.365455667	5500024030760072207028.A	.H07.G08.E09.D10.B11.A12
meclofenamic acid	34.74188851	35.10850208	-0.366613569	614615111406.A11	.H07.G08.E09.D10.B11.A12
proscillaridin	34.34077882	34.70962788	-0.368849062	5.50E+26	.H01.G02.E03.D04.B05.A06
sulfaquinoxaline	33.59180214	33.96076648	-0.36896434	5500024025833041107269.D	.H01.G02.E03.D04.B05.A06
josamycin	35.05567715	35.4281341	-0.372456952	612613111306.C10	.H07.G08.E09.D10.B11.A12
Prestwick-1084	36.87232086	37.24787327	-0.375552414	5500024035736031208612.H	.H01.G02.E03.D04.B05.A06
dicoumarol	33.57948872	33.96129529	-0.381806568	5.50E+33	.H07.E09.D10.B11.A12
sulfamethoxazole	33.07656102	33.45935203	-0.38279101	5500024024213121906560.H	.H01.G02.E03.D04.B05.A06
novobiocin	33.75035785	34.13330462	-0.382946763	5500024030760072207028.G	.H01.G02.D04.B05.A06
pentetic acid	33.82655991	34.21031976	-0.383759841	5500024030760072207028.D	.H07.G08.E09.D10.B11.A12
metronidazole	34.8823267	35.2669347	-0.384608008	612613111306.A04	.H01.G02.E03.D04.B05.A06
decamethonium brom	34.32476269	34.70962788	-0.38486519	5500024030760072207033.B	.H01.G02.E03.D04.B05.A06
valproic acid	34.0352853	34.42118584	-0.385900535	5.50E+26	.H01.G02.E03.D04.B05.A06
pivmecillinam	33.79033351	34.17633596	-0.386002446	5500024030760072207033.B	.H07.G08.E09.B11.A12
valproic acid	36.92306498	37.31290545	-0.38984047	5.50E+32	.G08.E09.D10.B11.A12
carbinoxamine	34.01052542	34.40088765	-0.390362233	5500024030401071707292.D	.H07.G08.E09.D10.B11
diflorasone	34.62349094	35.01997127	-0.396480325	5500024024213121906559.F	.H01.G02.E03.D04.B05.A06
tocainide	34.31297453	34.70962788	-0.396653354	5500024030760072207033.C	.H01.G02.E03.D04.B05.A06
trichostatin A	35.02689515	35.4281341	-0.401238946	612613111306.C11	.H07.G08.E09.D10.B11.A12
ebselen	33.99673574	34.40088765	-0.404151912	5500024030401071707292.F	.H07.G08.E09.D10.B11
ginkgolide A	36.43321091	36.83996258	-0.406751668	5.50E+32	.H07.G08.E09.D10.B11.A12
torasemide	33.76889588	34.17633596	-0.407440078	5500024030760072207033.F	.H07.G08.E09.B11.A12
trifluoperazine	32.59096302	33.00109826	-0.410135242	5.50E+31	.H07.G08.E09.D10.B11.A12
bromperidol	34.69770013	35.11129025	-0.413590117	640641112706.F04	.H01.G02.E03.D04.B05
pridinol	33.98196236	34.40088765	-0.418925291	5500024030401071707292.F	.H07.G08.E09.D10.B11
cefotetan	36.41761351	36.83996258	-0.422349068	5500024024213121906563.F	.H07.G08.E09.D10.B11.A12
chlorpromazine	33.99700466	34.42118584	-0.424181178	5500024030401071707292.F	.H01.G02.E03.D04.B05.A06
lisuride	35.00227256	35.4281341	-0.425861536	612613111306.A11	.H07.G08.E09.D10.B11.A12

fluvoxamine	34.28301494	34.70962788	-0.426612945	5500024030760072207033.F(	.H01.G02.E03.D04.B05.A06
aconitine	34.65720032	35.08636713	-0.429166808	640641112706.B09	.H07.G08.E09.D10.B11.A12
pentoxifylline	34.67524653	35.10850208	-0.433255543	614615111406.A10	.H07.G08.E09.D10.B11.A12
oxprenolol	36.81323908	37.24787327	-0.434634199	5500024035736031208612.D	.H01.G02.E03.D04.B05.A06
atovaquone	34.69634873	35.13162255	-0.435273827	5.50E+31	.H07.G08.E09.D10.B11.A12
tolfenamic acid	34.67171275	35.10850208	-0.436789324	614615111406.B08	.H07.G08.E09.D10.B11.A12
fenspiride	34.67046029	35.10850208	-0.438041783	6.15E+21	.H07.G08.E09.D10.B11.A12
nefopam	33.02105908	33.45935203	-0.438292952	5500024024213121906560.D	.H01.G02.E03.D04.B05.A06
lasalocid	33.69488816	34.13330462	-0.438416459	5500024030760072207028.A(	.H01.G02.D04.B05.A06
antimycin A	34.66740049	35.10850208	-0.441101589	614615111406.F07	.H07.G08.E09.D10.B11.A12
cefuroxime	33.51728164	33.96076648	-0.44348484	5500024025833041107269.D	.H01.G02.E03.D04.B05.A06
clotrimazole	33.00008867	33.4530122	-0.452923531	622623112706.H05	.H01.G02.E03.B05.A06
trichostatin A	36.385786	36.83996258	-0.454176577	5500024024213121906563.H	.H07.G08.E09.D10.B11.A12
cefaclor	34.67470972	35.13162255	-0.456912835	5500024024214122006604.D	.H07.G08.E09.D10.B11.A12
allantoin	37.18005661	37.63864853	-0.458591919	5500024024213121906564.G	.H07.G08.D10.B11.A12
dl-alpha tocopherol	36.38040487	36.83996258	-0.459557708	5500024024213121906563.F	.H07.G08.E09.D10.B11.A12
acetohexamide	37.17336593	37.63864853	-0.465282602	5500024024213121906564.A(	.H07.G08.D10.B11.A12
scopoletin	36.98993748	37.46016387	-0.470226386	5500024030700072107987.C	.H07.G08.E09.D10.B11.A12
valproic acid	33.94793719	34.42118584	-0.473248651	5500024030401071707292.G	.H01.G02.E03.D04.B05.A06
paclitaxel	34.9526173	35.4281341	-0.475516796	612613111306.A08	.H07.G08.E09.D10.B11.A12
hydroxyachillin	34.54331938	35.01997127	-0.476651892	5500024024213121906559.C	.H01.G02.E03.D04.B05.A06
memantine	34.22951189	34.70962788	-0.480115995	5500024030760072207033.B(	.H01.G02.E03.D04.B05.A06
gramine	34.53923521	35.01997127	-0.480736059	5.50E+22	.H01.G02.E03.D04.B05.A06
ambroxol	32.97592086	33.45797002	-0.482049156	622623112706.A07	.H07.G08.E09.D10.B11.A12
chlorprothixene	36.52375139	37.00786033	-0.484108942	5500024024213121906563.G	.H01.G02.E03.D04.B05.A06
cefalexin	36.51940747	37.00786033	-0.488452857	5500024024213121906563.F(	.H01.G02.E03.D04.B05.A06
trapidil	36.96949359	37.46016387	-0.490670279	5500024030700072107987.C	.H07.G08.E09.D10.B11.A12
wortmannin	33.92661721	34.42118584	-0.494568627	5500024030401071707292.A(	.H01.G02.E03.D04.B05.A06
trichostatin A	37.14399211	37.63864853	-0.494656417	5500024024213121906564.H	.H07.G08.D10.B11.A12
procainamide	36.50519985	37.00786033	-0.50266048	5500024024213121906563.H	.H01.G02.E03.D04.B05.A06
tetracycline	34.23448643	34.74789333	-0.513406898	614615111406.B03	.H01.G02.E03.D04.B05.A06
LY-294002	36.79636007	37.31290545	-0.516545382	5500024035736031208612.D	.G08.E09.D10.B11.A12
Prestwick-860	33.68668442	34.21031976	-0.52363534	5.50E+28	.H07.G08.E09.D10.B11.A12
suprofen	33.60480655	34.13330462	-0.528498063	5500024030760072207028.D	.H01.G02.D04.B05.A06
valproic acid	35.61074846	36.14209273	-0.531344272	5500024017802120306174.G	.H01.G02.E03.D04.B05.A06
LY-294002	33.88785705	34.42118584	-0.533328792	5500024030401071707292.B(	.H01.G02.E03.D04.B05.A06
15-delta prostaglandi	33.88547671	34.42118584	-0.535709127	5500024030401071707292.C	.H01.G02.E03.D04.B05.A06
procaine	37.08846715	37.63864853	-0.550181378	5500024024213121906564.G	.H07.G08.D10.B11.A12
phenformin	32.90840197	33.45935203	-0.550950059	5.50E+27	.H01.G02.E03.D04.B05.A06
mesalazine	36.692977	37.24787327	-0.554896276	5500024035736031208612.A(	.H01.G02.E03.D04.B05.A06
diprophylline	37.07781462	37.63864853	-0.56083391	5.50E+33	.H07.G08.D10.B11.A12
laudanosine	34.5467555	35.11129025	-0.564534752	640641112706.C06	.H01.G02.E03.D04.B05
Trolox C	34.54310794	35.11129025	-0.568182313	640641112706.D05	.H01.G02.E03.D04.B05
lansoprazole	33.60468108	34.17633596	-0.571654879	5500024030760072207033.C	.H07.G08.E09.B11.A12
xylometazoline	34.52454533	35.10850208	-0.583956747	6.15E+22	.H07.G08.E09.D10.B11.A12
troglitazone	33.82891193	34.42118584	-0.592273904	5500024030401071707292.C	.H01.G02.E03.D04.B05.A06
baclofen	34.82809146	35.4281341	-0.600042637	612613111306.C12	.H07.G08.E09.D10.B11.A12
sotalol	34.10250401	34.70962788	-0.60712387	5.50E+23	.H01.G02.E03.D04.B05.A06
risperidone	33.56792066	34.17633596	-0.608415299	5500024030760072207033.G	.H07.G08.E09.B11.A12
trimethobenzamide	34.65367669	35.2669347	-0.613258011	612613111306.A03	.H01.G02.E03.D04.B05.A06
norcyclobenzaprine	34.51078749	35.13162255	-0.620835062	5500024024214122006604.G	.H07.G08.E09.D10.B11.A12
cinoxacin	33.77856269	34.40088765	-0.622324954	5.50E+32	.H07.G08.E09.D10.B11
oxamniquine	34.07257057	34.70962788	-0.637057315	5500024030760072207033.D	.H01.G02.E03.D04.B05.A06
sulindac	36.99721672	37.63864853	-0.641431806	5500024024213121906564.D	.H07.G08.D10.B11.A12
securinine	33.75354916	34.40088765	-0.647338487	5500024030401071707292.C	.H07.G08.E09.D10.B11
acepromazine	34.43444908	35.08636713	-0.651918046	640641112706.C08	.H07.G08.E09.D10.B11.A12
harmine	34.42943532	35.08636713	-0.656931809	640641112706.G10	.H07.G08.E09.D10.B11.A12
ethosuximide	34.45075606	35.10850208	-0.657746015	614615111406.C10	.H07.G08.E09.D10.B11.A12
chlorphenesin	34.44596803	35.10850208	-0.662534049	614615111406.C09	.H07.G08.E09.D10.B11.A12
LY-294002	36.64945726	37.31290545	-0.663448195	5500024035736031208612.B	.G08.E09.D10.B11.A12
protriptyline	36.79527889	37.46016387	-0.664884983	5500024030700072107987.F	.H07.G08.E09.D10.B11.A12
valproic acid	35.46842782	36.14209273	-0.673664911	5500024017802120306174.A(	.H01.G02.E03.D04.B05.A06
flupentixol	36.33403983	37.00786033	-0.673820502	5500024024213121906563.D	.H01.G02.E03.D04.B05.A06
captopril	34.58962303	35.2669347	-0.677311679	612613111306.D06	.H01.G02.E03.D04.B05.A06
trichostatin A	34.05545584	34.74789333	-0.692437487	614615111406.A02	.H01.G02.E03.D04.B05.A06
harmol	34.41753298	35.11129025	-0.693757266	640641112706.A05	.H01.G02.E03.D04.B05
paracetamol	33.51340966	34.21031976	-0.696910099	5500024030760072207028.H	.H07.G08.E09.D10.B11.A12
15-delta prostaglandi	35.43906865	36.14209273	-0.703024077	5500024017802120306174.C	.H01.G02.E03.D04.B05.A06
valproic acid	35.43804264	36.14209273	-0.704050084	5500024017802120306174.F(	.H01.G02.E03.D04.B05.A06

mefenamic acid	36.93424583	37.63864853	-0.704402703	5500024024213121906564.C	.H07.G08.D10.B11.A12
trichostatin A	36.53196366	37.24471367	-0.712750013	5500024030700072107987.F	.H01.G02.E03.D04.B05.A06
ketoconazole	36.29077157	37.00786033	-0.71708876	5500024024213121906563.D	.H01.G02.E03.D04.B05.A06
apramycin	33.96989456	34.70962788	-0.739733323	5500024030760072207033.F	.H01.G02.E03.D04.B05.A06
amiodarone	34.3991054	35.15552717	-0.756421771	5500024024214122006604.F	.H01.G02.E03.D04.A06
valproic acid	36.52329317	37.31290545	-0.789612283	5500024035736031208612.F	.G08.E09.D10.B11.A12
LY-294002	33.62724215	34.42118584	-0.793943682	5500024030401071707292.D	.H01.G02.E03.D04.B05.A06
azathioprine	34.60302041	35.4281341	-0.825113691	612613111306.D09	.H07.G08.E09.D10.B11.A12
moroxydine	34.59662545	35.4281341	-0.831508647	612613111306.D08	.H07.G08.E09.D10.B11.A12
ellipticine	34.25374815	35.08636713	-0.832618974	640641112706.F12	.H07.G08.E09.D10.B11.A12
thiostrepton	34.29535691	35.13162255	-0.836265649	5500024024214122006604.H	.H07.G08.E09.D10.B11.A12
sulfaphenazole	36.79940102	37.63864853	-0.839247512	5500024024213121906564.H	.H07.G08.D10.B11.A12
pentetrazol	34.24705969	35.10850208	-0.861442385	614615111406.H12	.H07.G08.E09.D10.B11.A12
6-benzylaminopurine	32.59311787	33.45935203	-0.866234155	5500024024213121906560.D	.H01.G02.E03.D04.B05.A06
N-acetyl-L-leucine	36.37466345	37.24471367	-0.870050224	5.50E+27	.H01.G02.E03.D04.B05.A06
oxolinic acid	34.22589914	35.10850208	-0.882602933	614615111406.F12	.H07.G08.E09.D10.B11.A12
valproic acid	36.3909057	37.31290545	-0.921999757	5500024035736031208612.A	.G08.E09.D10.B11.A12
dequalinium chloride	36.04453234	37.00786033	-0.963327991	5500024024213121906563.F	.H01.G02.E03.D04.B05.A06
rosiglitazone	35.1615202	36.12983309	-0.968312888	EC2004032003AA	EC2004032002AA
etamivan	34.14048032	35.11129025	-0.970809931	6.41E+17	.H01.G02.E03.D04.B05
trichostatin A	33.44787813	34.42118584	-0.973307707	5500024030401071707292.C	.H01.G02.E03.D04.B05.A06
morantel	36.66372349	37.63864853	-0.97492504	5500024024213121906564.G	.H07.G08.D10.B11.A12
todralazine	36.65041631	37.63864853	-0.988232219	5500024024213121906564.G	.H07.G08.D10.B11.A12
tolnaftate	34.26373984	35.2669347	-1.003194866	612613111306.A02	.H01.G02.E03.D04.B05.A06
sodium phenylbutyrat	35.6465771	36.67804214	-1.031465036	EC2004042305AA	EC2004042302AA
chrysin	36.42113217	37.46016387	-1.039031699	5500024030700072107987.H	.H07.G08.E09.D10.B11.A12
hyoscyamine	34.03465862	35.10850208	-1.073843455	6.15E+23	.H07.G08.E09.D10.B11.A12
troglitazone	35.03613777	36.12983309	-1.093695327	EC2004032004AA	EC2004032002AA
verteporfin	36.15210901	37.24787327	-1.095764266	5500024035736031208612.F	.H01.G02.E03.D04.B05.A06
propafenone	33.99580021	35.11129025	-1.115490042	640641112706.F03	.H01.G02.E03.D04.B05
nifedipine	36.44715009	37.63864853	-1.191498437	5500024024213121906564.D	.H07.G08.D10.B11.A12
solanine	33.75689601	35.01997127	-1.263075263	5500024024213121906559.D	.H01.G02.E03.D04.B05.A06
parthenolide	33.80511904	35.11129025	-1.306171207	640641112706.C01	.H01.G02.E03.D04.B05
vorinostat	33.09407401	34.42118584	-1.327111825	5.50E+23	.H01.G02.E03.D04.B05.A06
apigenin	35.5107929	36.83996258	-1.329169676	5.50E+28	.H07.G08.E09.D10.B11.A12
rofecoxib	34.74809467	36.12983309	-1.381738421	EC2004032005AA	EC2004032002AA
estradiol	34.78813617	36.19585457	-1.4077184	EC2004032010AA	EC2004032009AA
wortmannin	34.70389207	36.19585457	-1.491962493	EC2004032012AA	EC2004032009AA
tetraethylenepentami	35.09089966	36.67804214	-1.587142485	EC2004042306AA	EC2004042302AA
raloxifene	34.58157554	36.19585457	-1.61427903	EC2004032011AA	EC2004032009AA
clopamide	31.71959007	33.45797002	-1.738379944	6.23E+22	.H07.G08.E09.D10.B11.A12
dirithromycin	33.28705768	35.11129025	-1.824232574	640641112706.H04	.H01.G02.E03.D04.B05
chlortetracycline	33.4197275	35.4281341	-2.008406601	612613111306.A07	.H07.G08.E09.D10.B11.A12
ajmaline	32.97105775	35.11129025	-2.140232496	640641112706.A04	.H01.G02.E03.D04.B05
trichostatin A	33.97743969	36.14209273	-2.164653037	5500024017802120306174.C	.H01.G02.E03.D04.B05.A06
azacyclonol	33.22679593	35.4281341	-2.201338173	612613111306.F12	.H07.G08.E09.D10.B11.A12
lobeline	32.87491587	35.08636713	-2.211451256	6.41E+23	.H07.G08.E09.D10.B11.A12
vorinostat	33.91319192	36.14209273	-2.228900814	5.50E+23	.H01.G02.E03.D04.B05.A06
glibenclamide	31.08342575	33.4530122	-2.369586453	622623112706.H02	.H01.G02.E03.B05.A06
clindamycin	32.36273132	34.74789333	-2.385162005	614615111406.F01	.H01.G02.E03.D04.B05.A06
chlorzoxazone	32.72003682	35.10850208	-2.388465254	614615111406.F09	.H07.G08.E09.D10.B11.A12
valproic acid	34.27194979	36.67804214	-2.406092354	EC2004042303AA	EC2004042302AA
ampyrone	32.33060324	34.74789333	-2.41729009	614615111406.A04	.H01.G02.E03.D04.B05.A06
trichostatin A	34.53995848	37.31290545	-2.772946968	5500024035736031208612.C	.G08.E09.D10.B11.A12
piperlongumine	32.25981733	35.08636713	-2.8265498	640641112706.F11	.H07.G08.E09.D10.B11.A12
danazol	32.54635143	35.4281341	-2.881782672	612613111306.B08	.H07.G08.E09.D10.B11.A12
colforsin	32.06885637	35.03613777	-2.9672814	EC2005081605AA	EC2005081602AA
valproic acid	33.10374681	36.67804214	-3.574295328	EC2004042304AA	EC2004042302AA
estradiol	31.08260455	35.03613777	-3.953533213	EC2005081604AA	EC2005081602AA
vorinostat	33.34232906	37.31290545	-3.970576396	5.50E+29	.G08.E09.D10.B11.A12
